# ACSS2/AATF Drives Soluble FasL‐Mediated CD8^+^ T Cell Apoptosis in Pancreatic Neuroendocrine Tumors

**DOI:** 10.1002/advs.202506883

**Published:** 2025-08-12

**Authors:** Qin Dang, Ting Wang, Yan Wang, Zeng Ye, Borui Li, Xuan Pan, Zheng Li, Guixiong Fan, Desheng Jing, Junfeng Xu, Qiangsheng Hu, Shunrong Ji, Xiaowu Xu, Xianjun Yu, Yi Qin

**Affiliations:** ^1^ Department of Pancreatic Surgery Fudan University Shanghai Cancer Center Shanghai 200032 China; ^2^ Department of Oncology Shanghai Medical College Fudan University Shanghai 200032 China; ^3^ Shanghai Pancreatic Cancer Institute Shanghai 200032 China; ^4^ Shanghai Key Laboratory of Precision Medicine for Pancreatic Cancer Shanghai 200032 China; ^5^ Pancreatic Cancer Institute Fudan University Shanghai 200032 China; ^6^ Department of Hepatopancreatobilary Surgery the First College of Clinical Medical Science Three Gorges University Yichang Hubei 443001 China; ^7^ The People's Hospital of China Three Gorges University Yichang Hubei 443001 China; ^8^ Department of Hepatobiliary Surgery Yijishan Hospital The First Affiliated Hospital of Wannan Medical College Wuhu 241001 China; ^9^ Department of Thoracic Surgery Shanghai Pulmonary Hospital Tongji University School of Medicine Shanghai 200433 China

**Keywords:** AATF, Acetyl‐CoA, ACSS2, Fas/FasL pathway, pancreatic neuroendocrine tumors

## Abstract

Besides the traditional carbon sources, Acetyl coenzyme A has recently been shown to be generated from acetate in various cancers, which subsequently promotes tumor growth and immune escape. However, the mechanism of Acetyl coenzyme A availability in pancreatic neuroendocrine tumors (PNETs) remains largely unknown. Herein, the metabolic‐epigenetic modification driven by acetyl coenzyme A synthase 2 (ACSS2) and its effect on the Fas/FasL system in PNETs is investigated. ACSS2 is highly expressed in PNETs and significantly correlated with patient prognosis. Mechanistically, ACSS2 activity or acetate supplementation induces histone H3/H4 hyperacetylated in PNET cells. This epigenetic modification recruits the transcription factor AATF to co‐regulate FasL transcription, specifically enhancing soluble FasL secretion. Secreted FasL binds Fas receptors on CD8^+^ T cells, activating caspase‐8/3 cascades to trigger T‐cell apoptosis and promote immune evasion. Notably, the finding indicated the non‐redundant and synergistic effects of ACSS2 and AATF in modulating FasL expression, which might support emerging strategies for immunotherapy of PNETs.

## Graphical abstract

This study unveils that ACSS2‐driven acetate metabolism fuels histone pan‐acetylation in pancreatic neuroendocrine tumors (PNETs), remodeling the epigenetic landscape. The ACSS2/AATF axis transcriptionally activates soluble FasL (sFasL), which engages Fas receptors on infiltrating CD8^+^ T cells to trigger caspase‐8/3‐mediated apoptosis. This metabolite‐epigenetic‐immune cascade promotes tumor immune evasion, thereby establishing ACSS2/AATF as a synergistic molecular nexus for targeted PNET immunotherapy.

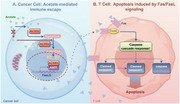

## 1 Introduction

Pancreatic neuroendocrine tumors (PNETs) are infrequent (account for ≈2% of all pancreatic tumors) and highly heterogeneous tumors, with clinical manifestations that associated with tumor progression and hormonal status, giving rise to a plethora of therapeutic options^[^ ^1^, ^2^ ^]^ Global NET registries document a rising incidence across all primary sites, disease stages, and histologic grades, with certain subtypes exhibiting up to 2.5‐fold increases over the past decade.^[^ ^3^ ^]^ In localized and metastatic G1/G2 PNETs, guideline‐endorsed systemic interventions spanning somatostatin analogs, radioligand therapy (^177^Lu‐DOTATATE), cytotoxic chemotherapy, and targeted agents (everolimus/sunitinib) redefined therapeutic benchmarks, delivering statistically significant and clinically relevant gains in outcomes.^[^ ^4^, ^5^, ^6^, ^7^, ^8^, ^9^ ^]^ Surveillance, Epidemiology, and End Results showed a main overall survival of 20 months in PNET patients with distant metastases.^[^ ^3^ ^]^ Recently, immunotherapy emerged as a new frontier in tumor therapy. In neuroendocrine tumors, advances in immunotherapy have shed light on new treatment options for patients. Hence, therapeutic response rates and targets for predicting responsiveness stand out as a prominent research focus.

Acetyl coenzyme A (acetyl‐CoA) is produced during nutrient catabolism to fuel the tricarboxylic acid cycle, attending profound and versatile roles.^[^ ^10^ ^]^ Acetyl‐CoA is also a substrate for lysine acetylation modification (an evolutionarily conserved mechanism) and is involved in regulating metabolism in response to nutrient availability and cellular metabolic status.^[^ ^11^, ^12^ ^]^ Clinical trials related to acetyl‐CoA metabolism in tumors have also been widely carried out (Table S1, Supporting Information). As a fundamental member of the acetyl‐CoA synthetase family, acetyl‐CoA synthetase 2 (ACSS2) is involved in energy metabolism and cellular acetylation by catalyzing the conversion of acetic acid to acetyl‐CoA.^[^ ^13^, ^14^ ^]^ ACSS2 is intimately implicated to tumorigenesis and progression.^[^ ^15^, ^16^ ^]^ Studies demonstrated that ACSS2 is responsible for the tumor immunosuppressive microenvironment through metabolic reprogramming of acetyl coenzyme A.^[^ ^17^, ^18^ ^]^ As proof, inhibition of ACSS2 could transform cancer cells from consumers to producers of acetic acid, releasing acetic acid that could be used as fuel by tumor‐infiltrating lymphocytes, enhancing the effects of T cells and thus promoting anti‐tumor response.^[^ ^17^ ^]^ Given the established role of ACSS2 in fueling histone acetylation via acetyl‐CoA synthesis,^[^ ^13^, ^14^, ^15^, ^16^ ^]^ and emerging evidence linking epigenetic reprogramming to T cell dysfunction,^[^ ^17^, ^18^ ^]^ we hypothesized that ACSS2‐driven histone hyperacetylation in PNETs might transcriptionally regulate immune relative checkpoints.

Fas, also termed as CD95, APO‐1, or TNFRSF6, is a cell death regulatory receptor belonging to the tumor necrosis factor receptor family that triggers apoptosis.^[^ ^19^, ^20^ ^]^ The physiological ligand for FAS, Fas Ligand (FasL, also known as CD95L), is a member of the corresponding TNF cytokine family, and the membrane‐bound and soluble forms of the Fas/FasL system are major players in the activation of the death structural domain and in caspase 8/3 cleavage.^[^ ^21^ ^]^ The Fas/FasL pathway plays a crucial role in tumor immunity, especially in the clearance of pathogen‐infected target cells and in the death of no longer needed, potentially harmful, and autoreactive lymphocytes.^[^ ^22^, ^23^ ^]^ This ligand‐receptor pair thus serves as a guardian of autoimmunity and tumor development.^[^ ^24^, ^25^ ^]^ In tumor immunity, Fas/FasL mediates off‐target “bystander” killing of antigen‐negative tumor cells.^[^ ^24^ ^]^ Ovarian cancer patients highly express FasL and mediate immune escape.^[^ ^26^ ^]^ Low‐dose doxorubicin in combination with sodium nitroprusside could induce sustained production of nitric oxide, which in situ upregulated the expression of Fas on the surface of tumor cells, thereby sensitizing the Fas/FasL pathway‐mediated apoptosis of tumor cells.^[^ ^27^ ^]^ The direct link between the Fas/FasL pathway and acetyl‐CoA metabolism has not yet been clarified, but it has been shown that metabolic modulators, such as histone deacetylase inhibitors, could upregulate the expression of Fas on tumor cells, thereby affecting the ability of osteosarcoma to form lung metastases.^[^ ^28^ ^]^ This implicates acetyl‐CoA metabolism may drive tumor progression and metastasis through the Fas/FasL system.

To sum, while acetyl‐CoA‐dependent transcriptional regulation is well‐established, how metabolic enzymes discretely govern immune checkpoints in PNETs remains elusive. Herein, this study identifies the ACSS2/AATF axis as a compartment‐specific regulator of sFasL—a bridge coupling tumor metabolic reprogramming to CD8⁺ T cell exhaustion. Critically, pharmacologic disruption of this axis using ACSS2 inhibitors or FasL‐blocking antibodies demonstrates target engagement and functional efficacy, serving actionable targets for PNETs immunotherapy.

## 2 Experimental Section

### 2.1 Clinical Populations and Information Collection

105 patients clinically diagnosed with PNETs and receiving surgical treatment were enrolled and followed up at Fudan University Shanghai Cancer Center (FUSCC) from June 2012 to January 2023. 105 paired cancer and adjacent tissues were constructed into tissue microarrays (TMAs). Tables S2 and S3 (Supporting Information) provide the record of the baseline data.

For a total of nine fresh patient tissue specimens (enrolled at FUSCC from March 2024 to May 2024) that were to be subjected to primary cell isolation and culture, the tumor tissue block was processed by a pathologist under sterile conditions into 8–10 mm^3^ blocks after surgical resection to avoid areas of necrosis. All patients gave informed consent, and the ethics committee of the Fudan University Shanghai Cancer Center provided consent to the study. The study was performed in accordance with the Declaration of Helsinki.

### 2.2 Cell Culture and Primary Cell Isolation

Human PNET cell line BON‐1 was granted by Professor Martyn Caplin (Professor of Gastroenterology & Gastrointestinal Neuroendocrinology Centre for Gastroenterology, Royal Free Hospital, London). Human PNET cell line QGP‐1 was purchased from Shanghai Zhong Qiao Xin Zhou Biotechnology and identified by short tandem repeat sequence. BON‐1 was cultured in DMEM/F12 and QGP‐1 was cultured in RPMI 1640 medium, both containing 100U mL^−1^ penicillin and 100 mg mL^−1^ streptomycin solution and supplemented with 10% fetal bovine serum (FBS) (Biochrom, Holliston, MA, USA), in a 5% CO2 and 95% air incubator at 37 °C. The Jurkat cell line was from the National Collection of Authenticated Cell Cultures and grown in standard conditions. Dimethyl sulfoxide (DMSO, D2650, Sigma–Aldrich), acetate (S8750, Sigma–Aldrich), ACSS2 inhibitor (ACSS2i, vy‐3‐135, #1824637‐41‐3, MedChemExpress), FasL blocking antibody (FasL antibody) (ab186671), animal‐free recombinant human soluble Fas ligand (rh‐sFasL, HY‐P700052AF, MedChemExpress), and Kp7‐6 (HY‐P10102, MedChemExpress) follow the manufacturer's instructions. Positive clones were selected in puromycin (Gibco)‐containing medium. Mycoplasma tests every two weeks were negative.

For primary cell extraction, 8–10 mm^3^ blocks of fresh tissue obtained as described above were cut into ≈1 mm^3^ pieces, and the tissue blocks were washed with pre‐cooled Dulbecco's phosphate‐buffered saline (DPBS, ThermoFisher Scientific). The tissues were subsequently digested in 10 mL of digestion medium (including 1 mg mL^−1^ collagenase IV and RPMI‐1640 medium) in a 37 °C incubator for 6–8 h, and the digestion medium was blown with a pipette gun once an hour to fully separate the cells from the connective tissue. The filtrate was collected after two filtrations with 70 µm filters (corning, 431 751, US). Erythrocytes were lysed with ACK lysis buffer (Gibco, A10492, US) at room temperature. After centrifugation at 180 g for 5 min and aspiration of the supernatant, cells were resuspended in complete medium and counted. Subsequently, human CD45^+^ cells in tissue single cell suspensions were isolated and purified by EasySep Magnetic Bead Enrichment Kit (STEMCELL Technologies, 100–0105, Canada) for initial enrichment of leukocytes. Further, T cells expressing CD8 molecules on their surface were labeled with specific antibodies and sorted by flow cytometry.

Prior to acetate (1 mm) treatment, cells were cultured in low glucose medium (5 mm) supplemented with dialyzed FBS (30 067 334, Thermo Fisher Scientific), 100U mL^−1^ penicillin, and 100 mg mL^−1^ streptomycin solution.

### 2.3 Co‐Immunoprecipitation and Mass Spectrometry Analysis

For identification of interacting proteins, whole‐cell extracts were obtained using label‐free quantification for BON‐1 cells with stable Flag‐ACSS2 expression on ice with pre‐cooled NP‐40 lysis buffer (Beyotime) at 4 °C, following which the extracts were used to obtain MS uptake samples according to the Flag‐tag Protein IP Assay Kit with Magnetic Beads kit (P2181S, Beyotime). The Mass spectrometry (MS) detection and analysis procedure was the same as the previous study.^[^ ^29^ ^]^ Meanwhile, whole‐cell extract was subjected to gel electrophoresis and staining with a silver staining kit (Beyotime).

### 2.4 Western Blot and Quantitative Real‐Time PCR

Cell lysates were prepared from RIPA lysis buffer (P0013B, Beyotime), deacetylase inhibitor cocktail (HY‐K0030, MedChemExpress), protesase and phosphatase inhibitors (MedChemExpress) were prepared for western blot analysis using antibodies (Table S4, Supporting Information). β‐actin serves as the loading control.

The RNA was extracted by RNAiso Plus (Takara) reagent, and the quality was evaluated by NanoDrop One C (Waltham, USA). Complementary DNA (cDNA) was obtained by following the protocol of the Reverse Transcription Kit (Takara). Then, quantitative real‐time polymerase chain reaction (qRT‐PCR) was performed using ABI 7900HT Fast system and SYBR Green PCR Master Mix (Yeason). Each test was repeated three times. The expression level was quantized by 2^‐ΔΔCt^ mode. β‐actin serves as an internal reference for normalization. The detailed information for the primer sequence is referred to Table S5 (Supporting Information).

### 2.5 Immunohistochemical Staining and Multiplex Immunofluorescence

TMA blocks containing areas of tumor tissue and adjacent areas of normal tissue were used for immunohistochemical (IHC) staining for ACSS2, ACLY, ACSS1, and PDH. The IHC staining procedure was as described previously.^[^ ^29^ ^]^ Multiplex immunofluorescence was performed on formalin‐fixed, paraffin‐embedded sections using the Opal 6‐Plex Manual Detection Kit (NEL811001KT, Akoya Biosciences). Antibody specificity was confirmed by peptide blocking assays.^[^ ^30^ ^]^ Optimal antigen retrieval (pH 9.0 Tris‐EDTA), autofluorescence quenching (0.3% Sudan Black B), and spectral unmixing parameters were established to minimize crosstalk (<1.5%) and maximize signal‐to‐noise ratio (Dice coefficient >0.92).^[^ ^31^ ^]^ Further, the specimens were divided into 5 groups: the weakest group (±, 0% to 1%), weak group (+, 2 to 10%), medium group (++, 11% to 30%), strong group (+++, 31 to 70%), and strongest group (++++, 71% to 100%). The ±, +, and ++ groups were further subdivided into low‐expression groups (0 to 30%) and the +++ and ++++ groups into high‐expression groups (31 to 100%). Tyramide signal amplification ‐based multiplex immunohistochemical (mIHC) was used to in situ label ACSS2 and FasL on tissue samples and to detect the correlation between their expression. Scoring of the tissue sections was performed by at least two senior pathologists independently.

### 2.6 Plasmid Transfection and RNA Interference

The pLKO.1 TRC cloning vector (Addgene plasmid # 10878) was used to knock down ACSS2 and AATF, targeting two sequences for each as follows:

ACSS2‐KO1: 5′‐CTGGCTATGGTACCACCGGG‐3′;

ACSS2‐KO2: 5′‐GGAACCAAGGGATTGACTTG‐3′;

AATF‐KO1: 5′‐CACCGTCCTTCGAGAACTCATAGAA‐3′;

AATF‐KO2: 5′‐CACCGAACTGGCTTCTGGAAAACTG‐3′.

The coding sequence of human ACSS2 and AATF was cloned into the lentiviral vector pCDH‐CMV‐MCS‐EF 1‐puro (SBI, USA) to generate the ACSS2 or AATF expression plasmid. Relative knockdown‐cell and overexpression‐cell pools were selected with puromycin after viral transduction. The empty vector (EV) serves as the reference for the overexpression‐cell pool, and the control (Ctrl) serves as the reference for the knockdown‐cell pool.

Small interfering RNAs (siRNAs) (MedChemExpress) were transiently transfected with Lipofectamine 3000 reagent (Life Technologies) and followed the manufacturer's instructions.

### 2.7 Enzyme‐Linked Immunosorbent Detection and Caspase‐8/3 Activity Assay

The content of acetyl‐CoA and soluble FasL (sFasL) was measured using corresponding Enzyme‐linked immunosorbent (ELISA) kits according to the manufacturer's instructions. Acetyl‐CoA content was calculated from the Acetyl‐CoA Content Assay Kit (BC0980, Solarbio). Human CD178 /FasL ELISA Kit was purchased from Diaclone Research, Besancon (#850.750.096). The detection limit of sFasL was 12 pg mL^−1^. Five independent repetitions were made for each sample assay. Fluorescence‐based assays regarding the activity of caspase‐8 and caspase‐3 were performed by Fluorometric Caspase‐8 Activity Assay Kit (#ab39534) and Fluorometric Caspase‐3 Activity Assay Kit (#ab39383) purchased from Abcam.

### 2.8 Chromatin Immunoprecipitation (ChIP)‐re‐ChIP Assay and Dual‐Luciferase Reporter Gene Assay

The BON‐1 and QGP‐1 cells were transfected with a Flag‐ACSS2 plasmid (GeneChem, Shanghai, China) and then subjected to ChIP‐re‐ChIP assay. ChIP assay was performed using the SimpleChIP plus Sonication Chromatin IP Kit (9003S, Cell Signaling Technology) according to the manufacturer's instructions. The primers were synthesized according to the AATF binding site of the *FasLG* promoter. Primer sequences for PCR were as follows: FasLG‐Forward: 5′‐ATTGTGGGCGGAAACTTC‐3′; FasLG‐Reverse: 5′‐ TCTCTGATTCTGCTTCTCAA‐3′. ChIP‐re‐ChIP was performed as described previously.^[^ ^32^ ^]^ Briefly, cross‐linked chromatin was sonicated into fragments, which were then immunoprecipitated using AATF or Flag antibodies. The immunoprecipitated complexes were eluted with re‐ChIP buffer.

BON‐1 cells overexpressing AATF were grown in 96‐well plates, and the FasLG promoter region spanning −2000 to +200 across the transcription start site was cloned into the pGL3‐Basic vector. After 24 h of incubation, cells were harvested, and luciferase activity was measured.

### 2.9 Immunofluorescence Staining and Confocal Microscope

Cells totaling 5 × 10^4^ were inoculated in Thermo Scientific Nunc Glass Bottom Dishes (150682) and cultured until apposition. After the designated treatments, cells were fixed with 4% paraformaldehyde and permeabilized by 0.25% Triton X‐100 at room temperature. After incubation with primary antibody and fluorochrome‐conjugated secondary antibody, the fluorescence intensity of the cells was observed by Leica SP5 II confocal laser scanning microscope (Leica, Wetzlar, Germany), and representative cells were selected and photographed.

### 2.10 Surface Plasmon Resonance (SPR)

Recombinant human ACSS2 and AATF binding kinetics were measured at 25 °C on a Biacore 8K SPR instrument (Cytiva). Experiments were performed by Shanghai SynSun Biotechnology Co., Ltd. A running buffer containing HBS‐EP was prepared, filtered, and degassed before use. Flow cells of a CM5 sensor chip were activated with a 1:1 mixture of 0.1 m N‐hydroxysuccinimide and 0.1 M N‐ethyl‐N’‐(3‐diethylaminopropyl)‐carbodiimide at a flow rate of 10 µL min^−1^. The ACSS2 were diluted in 10 mM sodium acetate (pH 5.5) to a concentration of 5 µg mL^−1^, and immobilized on the CM5 chip to a response signal. The remaining binding sites on the chip were blocked by 1 m ethanolamine (pH 8.5). Indicated AATF at a series of concentrations was injected into the flow cell to pass over the immobilized target. The binding signal was measured by a multiple‐cycle method with 120 s association and 120 s dissociation at a flow rate of 30 µL min^−1^. The AATF concentrations are 12.5, 25, 50, 100, 200, and 400 nm. The equilibrium dissociation constants (binding affinity, K*_D_*) of the interaction were calculated using BIAcore 8K evaluation software.

### 2.11 Propidum Iodide Staining and Fluorescence‐Activated Cell‐Sorting Analysis

Annexin V and Propidum iodide (PI) staining, as well as fluorescence‐activated cell‐sorting (FACS) analysis, were widely applied for the determination of cellular death through apoptosis. Cell samples were fixed in 70% ethanol solution overnight at 4 °C, then stained with PI (36 µg mL^−1^, Sigma) containing RNase (10 µg mL^−1^, Sigma) for 30 min at 37 °C, and finally analyzed for cell‐cycle profiles by CyAn ADP (Beckman Coulter). Each detection was performed independently with 3 replications.

### 2.12 Single‐Cell Sequencing and Data Analysis

In this study, single‐cell sequencing (scRNA‐seq) data were collected and analyzed with 15 pancreatic neuroendocrine tumor patients who underwent surgical operation at FUSCC. Ethical approval was confirmed by the Ethics Committee of the FUSCC (NO. 2105235–9), and all patients had written informed consent. Single‐cell RNA sequencing (scRNA‐seq) and V(D)J libraries were constructed using the Chromium Controller Instrument with the Chromium Single Cell 5′ Library & Gel Bead Kit and V(D)J Enrichment Kit (10x Genomics, v2 chemistry).^[^ ^33^, ^34^, ^35^ ^]^ The details of single‐cell dissociation and single‐cell sequencing were described in the previous research.^[^ ^36^ ^]^ Briefly, viable cells were concentrated to 1 × 10^3^ cells µL^−1^, and ≈10000 cells per channel were partitioned into Gel Bead‐in‐Emulsions (GEMs) to achieve targeted mRNA barcoding of 6000 single cells per sample. Following reverse transcription (Maxima H Minus Reverse Transcriptase, Thermo Fisher), GEMs were chemically disrupted, and cell‐barcoded full‐length cDNA was purified (SPRIselect, Beckman Coulter) followed by 12‐cycle PCR amplification. Amplified cDNA was split for parallel construction of: i) 5′gene expression libraries through enzymatic fragmentation (Tn5 transposase), A‐tailing, adapter ligation, and 14‐cycle index PCR; ii) TCR/BCR‐enriched libraries via V(D)J‐specific primer extension (10x Immune Profiling Solution). Library quantification was performed using Qubit dsDNA HS Assay (Thermo Fisher, Q33231), and size distribution was assessed with Agilent 2200 TapeStation (High Sensitivity D5000 ScreenTape, 5067–5592). All libraries underwent 150‐bp paired‐end sequencing on Illumina NextSeq 2000 (P3 flow cell) targeting 50000 reads per cell.

The scRNA‐seq data processing pipeline employed the Seurat R package Seurat (version 4.4.0). Potential doublets were detected and eliminated with DoubletFinder. For quality control, the cells with fewer than 200 detected genes or more than 40% mitochondrial content were removed to avoid interference with the analysis. The cell‐level normalization was performed by the LogNormalize method, and the top 2000 highly variable genes were identified using the FindVariableFeatures function. Data dimensionality reduction was used for principal component analysis (PCA) with 30 components. The cell clusters were identified and visualized with 0.4 resolution using the uniform manifold approximation and projection (UMAP) algorithm based on FindNeighbors and FindClusters functions. The initial round of cell clustering and annotated each subpopulation with known markers were performed as following: B cells (CD79A, MS4A1, CD19), T cells (CD3D, CD3E, GNLY), Mast cells (MS4A2, TPSB2, TPSAB1), Fibroblasts (ACTA2, COL1A2, COL3A1), Ductal cells (INS, PRSS1, CHGA, CHGB), Acinar cells (CPA1, CELA3B, PRSS1, CELA3A), Endothelial cells (CHGB, CHGA, ALDH1A1, EPCAM), Endothelial cells (PECAM1, VWF, ENG), Macrophages (CD68, CD163, CD14), and Cycling cells (MKL67, STMN1, TOP2A). To distinguish T cell subpopulations, the same approach and pipeline were further performed. The CD4, CD8, CD3D, CD3E, and FOXP3 were used to identified CD4^+^ T cells, CD8^+^ T cells, and Tregs. Meanwhile, the statues of T cell subpopulations were distinguished as naïve (LEF1, SELL, TCF7, CCR7, LTB, and KLF2) and cytotoxicity (IFNG, GZMB, PRF1, GZMA, and GZMH). Continuous variables were compared among multiple groups through the Kruskal‐Wallis test. *P*<0.05 was considered significant.

### 2.13 Patient‐Derived Organoid (PDO)

Three established PNET patient‐derived organoids (PDOs) following the previously developed procedure were used for analyses.^[^ ^37^ ^]^ Briefly, tissues were minced and incubated in digestion medium (2.5 mg mL^−1^ collagenase II and 10 µm Y‐27632 in basic medium) at 37 °C with mild agitation for 1 h. The obtained cells were inoculated on suspension plates with Matrigel and cultured with basic medium (advanced DMEM/F12, 10 mM HEPES, 1X GlutaMAX‐I, 100 µg mL^−1^ Primocin, 1X penicillin/streptomycin solution) and complete medium (advanced DMEM/F12, 10 mM HEPES, 1X GlutaMAX‐I, 100 µg mL^−1^ Primocin, 1X penicillin/streptomycin solution, 500 nm A83‐01, 10 µm Y‐27632, 1.56 mm N‐acetylcysteine, 10 mm nicotinamide, 10 ng mL^−1^ FGF10, 1X B27 supplement, 10 µm forskolin, 30% Wnt3A conditioned medium, 2% R‐spondin conditioned medium, 4% Noggin conditioned medium). The media used for organoid cryopreservation were composed of the corresponding culture medium (90%) and 10% DMSO. The previously developed PDOs showed extensive neuroendocrine features, including islet morphology and positive staining of synaptophysin (SYN) and CGA in organoid culture.^[^ ^37^ ^]^

### 2.14 TCR‐Activated CD8+ T Cells and Human Interferon Gamma (IFN‐γ) ELISPOT Assay

CD8^+^ T cells were stimulated with αCD3/CD28 Dynabeads (11131D, Thermo Fisher Scientific) at a 1:1 ratio in the presence of 30 U mL^−1^ recombinant Human IL‐2 (202IL050CF, R&D system) for 72 h at 37 °C in 5% CO_2_.^[^ ^38^ ^]^ The activated CD8^+^ T cells were co‐cultured with the organoid model in groups according to Figure S5C (Supporting Information) for 48 h. Group I added with 50 ng mL^−1^ hTGF‐β (100‐21, PeproTech) was listed as a negative control group.^[^ ^39^, ^40^ ^]^ Group I served as blank control groups. The culture plate was a 96‐well plate from the human interferon gamma (IFN‐γ) ELISPOT assay kit (ESP‐H0002, Elabscience). The ELISPOT assays were then performed according to the manufacturer's instructions. In short, ≈1 × 10^5^ per well of the co‐cultured CD8^+^ T cells were inoculated into 96‐well plates pre‐coated with anti‐human IFN‐γ monoclonal antibody at the bottom, and cultured at 37 ° C for 24 h. Plates were scanned using the S6 ultra immunoscan reader (Cellular Technology Ltd).

### 2.15 Mice

Animal maintenance and all experiments were performed in accordance with the National Institutes of Health Guide for the Care and Use of Laboratory Animals, and the institutional Animal Care and Use Committee (IACUC), and approved by the Shanghai Experimental Animal Center (Shanghai, China) (NO. FUSCC‐IACUC‐2024431 and FUSCC‐IACUC‐2025026). Mice were maintained in an air‐conditioned room (22 ± 2 °C) under a 12 h/12 h light/dark cycle (lights‐on at 7:00 am) and allowed water and standard chow.

MEN1^flox/flox^ (MEN1^f/f^) mice were generated by CRISPR/Cas 9‐stimulated homologous recombination. To generate mice with MEN1 deletion specifically in the pancreas, MEN1^f/f^ mice were crossed with Pdx1‐Cre transgenic mice (strain NO: T004860, GemPharmatech, Nanjing, China). All mice were crossed on a C57BL/6 background for at least three generations. The genotype of MEN1^f/f^, Pdx1‐Cre +, and MEN1‐conditional knockout (*Men1‐*KO) mice was confirmed by PCR using specific primers as follows: Pdx1‐Cre (Forward, 5′‐GGGCAGTCTGGTACTTCCAAGCT‐3′; Reverse, 5′‐TTCTGGGCTATACAAGCATCTGC‐3′) and MEN1 floxed alleles (Forward, 5′‐TAAACCTTGTGTGGTGGGGCAG‐3′; Reverse, 5′‐ CTTTGTCCTTAGTCAAGCCCGTG ‐3′).

Based on the reported similarity between the development of spontaneously formed pancreatic tumors in Rip1‐Tag2 PNET‐transgenic mouse model and human pancreatic neuroendocrine tumors, this animal model was obtained and successfully constructed from Professor Qi's laboratory.^[^ ^41^, ^42^, ^43^ ^]^ All animal experiments were approved by the internal animal protocol review committee. Mice were sacrificed in a carbon dioxide chamber at the end of the study. From the 12^th^ week, the ACSS2i group was intraperitoneally injected daily with vehicle (10% DMSO) of 100 mg kg^−1^ VY‐3‐135, the Kp7‐6 group was intraperitoneally injected daily with vehicle (10% DMSO) of 100 mg kg^−1^ Kp7‐6, and the control mice received an injection of an equivalent volume of vehicle alone. Lab members were not blinded to the treatment groups.

### 2.16 Statistics and Reproducibility

The study data were performed in R software (4.1.0), ImageJ (2.14.0), Graphpad Prism (10.0), and SlideViewer (2.5.0). Pearson's chi‐square test was used for categorical variables. Continuous variables were compared by adapting the Wilcoxon rank‐sum test or Student's t test. The survival curve was plotted using the Kaplan–Meier (K–M) method and compared using the log‐rank test. All data were presented as mean ± SD of at least three independent experiments. Two‐tailed Student's *t*‐test was used for pairwise comparisons unless otherwise noted. Pearson's correlation coefficient was used to measure the strength and direction of the linear relationship between two continuous variables. *P*‐values less than 0.05 were considered significant.

### 2.17 Ethics Declarations

The study was approved by the Ethics Committee of Fudan University Shanghai Cancer Center (2105235‐9), and written informed consent was obtained from each patient.

## 3 Results

### 3.1 The Expression of ACSS2 was Highly Elevated in PNETs and Predicts Unfavorable Outcomes

**Figure 1:**
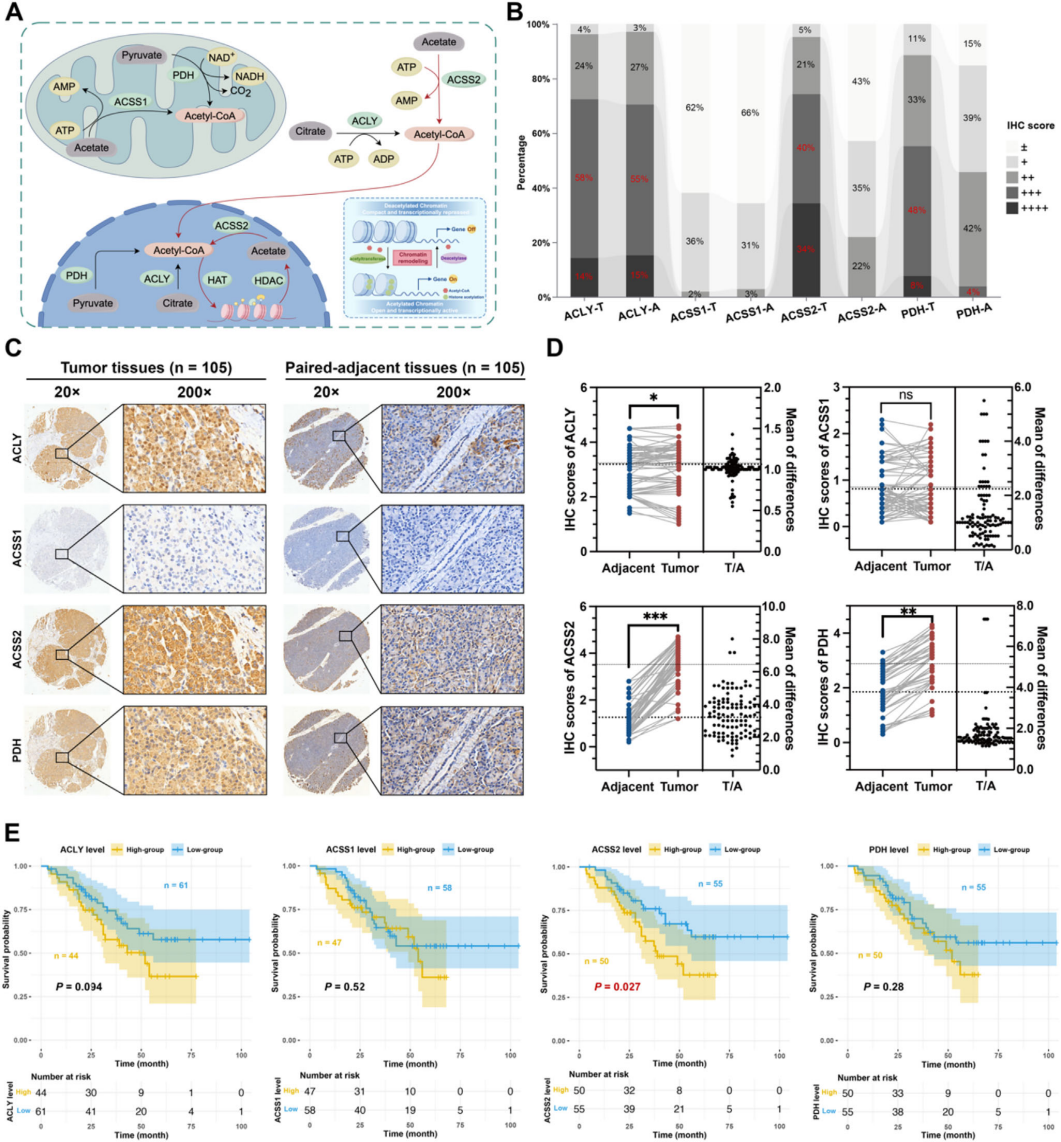
The expression level of ACSS2 was highly elevated in PNETs and predicts poor outcomes. A) Diagram of the major pathways of intracellular acetyl‐CoA anabolism and the dynamically regulated pattern of acetylation modifications occurring in histones. Intracellular sites of acetyl‐CoA anabolism and products of ACLY (substrate: citrate), ACSS1 (substrate: acetate), ACSS2 (substrate: acetate), and PDH (substrate: pyruvate). B) Immunohistochemistry (IHC) staining of human PNET tissues and paired adjacent normal tissues arrays using ACLY, ACSS1, ACSS2, and PDH‐specific antibodies (n = 105). C) Classification of tumor tissues (T) and adjacent normal tissues (A) according to the staining intensity of ACLY, ACSS1, ACSS2, and PDH (n = 105). D) ACLY, ACSS2, and PDH expression were significantly higher in PNET tissues than in paired adjacent normal tissues in IHC staining of tissue arrays, while there was no significance in the expression of ACSS1 expression between tumor tissues and paired adjacent normal tissues (^*^ *p* < 0.05, ^**^ *p* < 0.01, ^***^ *p* < 0.001, ns indicates not significant, n = 105). E) Kaplan‐Meier analysis of the overall survival rate of patients with PNET according to the expression of ACLY, ACSS1, ACSS2, and PDH, respectively (n = 105, log‐rank test).

Histone acetylation is the process of transferring the acetyl group of acetyl‐CoA to histone lysine residues in the presence of acetyltransferases.^[^ ^44^ ^]^ Acetyl‐CoA synthetase completes the synthesis of acetyl‐CoA by catalyzing the substrate acetate, which provides the necessary raw material for histone acetylation.^[^ ^13^, ^45^ ^]^ This post‐translational modification of histones increases chromatin accessibility and regulates gene expression (**Figure** **1**A).^[^ ^44^, ^46^ ^]^ Given that acetyl‐CoA is an important substrate for histone acetylation modifications to occur and its reported important role in tumorigenesis and development, the present study looked at the expression levels of representative synthases that could catalyze the dynamic production of acetyl‐CoA, ACLY, ACSS1, ACSS2, and PDH, in patients with PNETs (Figure 1A).^[^ ^10^, ^47^ ^]^ IHC assays in the tissues of 105 paired PNETs patients showed that the basal expression of ACLY and ACSS2 was higher than that of ACSS1 and PDH (Figure 1B,C). Further, IHC scoring showed that the expression of ACLY, ACSS2, and PDH was higher in tumor tissues than in adjacent normal tissues, and there was no linear correlation between the expression level of ACLY, ACSS1, ACSS2, and PDH (Figure 1D; Figure S1A, Supporting Information). To assess the impact of the expression of ACLY, ACSS1, ACSS2, and PDH on the prognosis of patients, we performed a Kaplan–Meier (K–M) survival analysis on 105 patients with PNETs with complete follow‐up data and categorized them into high‐ and low‐expression groups based on the median expression level of each molecule. The K–M curves showed that the expression of ACSS2 levels, but not the other 3 key enzymes, showed a statistically significant correlation with patient prognosis (Figure 1E). Patients in the high ACSS2 group showed a high clinical grade and an earlier decline in the survival curve, which was significantly lower than that of the patients with low ACSS2 expression (Figure S1B, Supporting Information). In closing, ACSS2's dominance over other acetyl‐CoA synthases positions it as the primary epigenetic modulator in PNETs.

### 3.2 ACSS2 Catalyzes Histone Hyperacetylation to Drive FasL Transcription in PNET Cells

**Figure 2:**
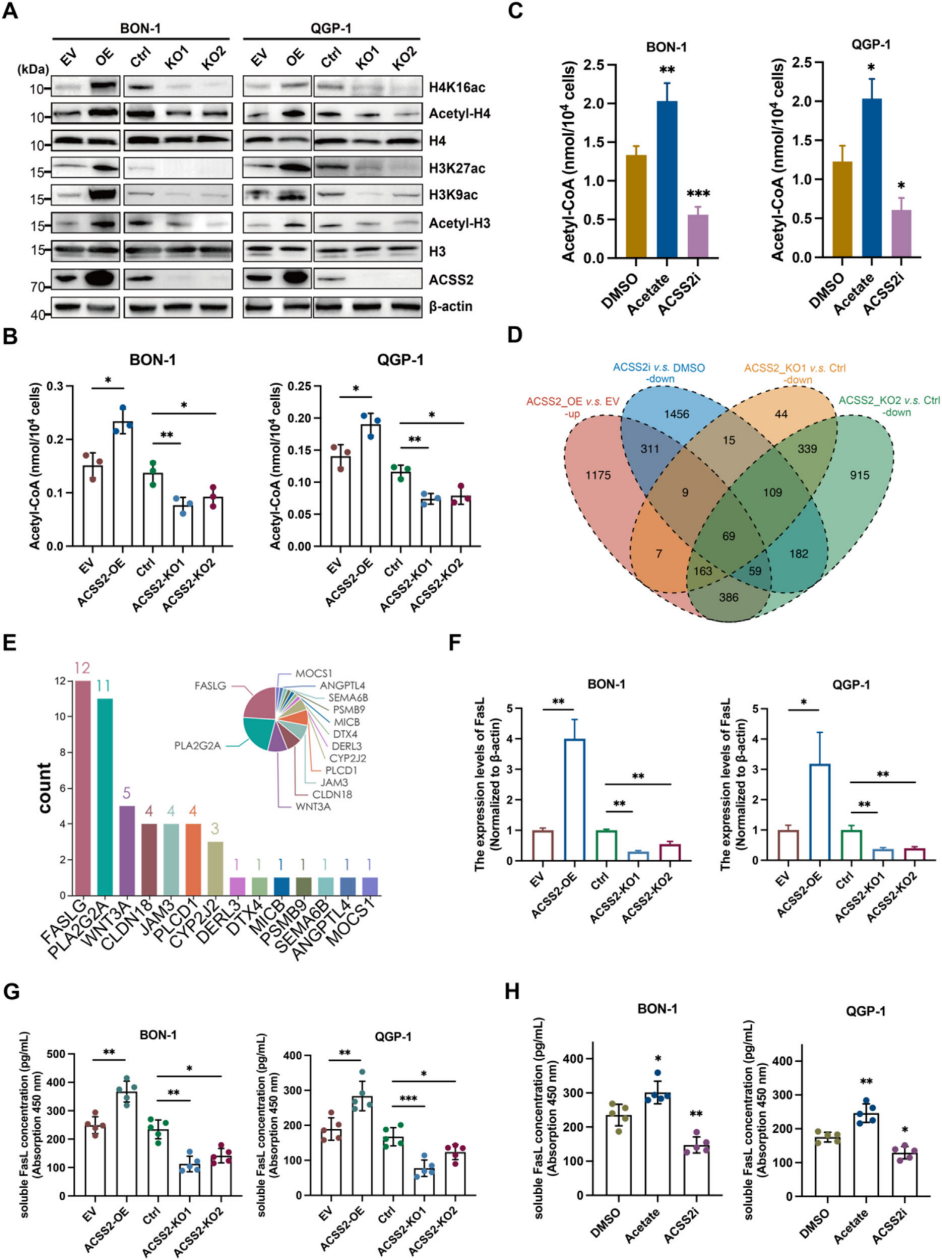
Increased ACSS2 catalyzes PNETs cells histone acetylation modification through pan‐acetylation regulation, and promotes FasL transcription and exocytosis of sFasL in PNET cells. A) ACSS2 enhances histone pan‐acetylation in PNET cell lines. After overexpression or knockdown of ACSS2, immunoblotting was performed with an antibody against ACSS2, using β‐actin as the loading control, and was performed with the antibodies against Ace‐H3, Ace‐H4, H3K9ac, H3K27ac, and H4K16ac, using H3 or H4 as the loading control. B) ACSS2 was overexpressed or knocked down in PNET cell lines, and acetyl‐CoA content was determined by fluorescence ELISA. The assay was repeated independently 3 times (^*^ *p* < 0.05, ^**^ *p* < 0.01). C) Cellular acetyl‐CoA content assay in BON‐1 and QGP‐1 cells after treatment with acetate supplementation or ACSS2i, respectively. D) Cross‐analysis of differentially expressed genes in the four comparison pairs (ACSS2‐OE vs EV‐up, ACSS2‐KO1 vs Ctrl‐down, ACSS2‐KO2 vs Ctrl‐down, ACSS2i vs DMSO‐down) yielded a total of 69 common genes. E) Ranking of the total number of pathways enriched for common genes. The vertical coordinate is the total number of pathways enriched to, and the horizontal coordinate is the gene name. F) *FasLG* gene expression levels were analyzed at the RNA level as verified by RT‐qPCR assays in different treatment groups of ACSS2 in BON‐1 and QGP‐1 cell lines, respectively (^*^ *p* < 0.05, ^**^ *p* < 0.01, ^****^ *p* < 0.0001). G) ELISA assay of soluble FasL (sFasL) levels in BON‐1 and QGP‐1 cell culture supernatants from overexpression or silencing of ACSS2. Five independent repetitions for each group (^*^ *p* < 0.05, ^**^ *p* < 0.01, ^***^ *p* < 0.001). H) ELISA assay of sFasL levels in BON‐1 and QGP‐1 cell culture supernatants from different treatment groups. Five independent repetitions for each group (^*^ *p* < 0.05, ^**^ *p* < 0.01).

As a key enzyme in acetyl‐CoA biosynthesis, ACSS2 demonstrates significant upregulation in PNET and correlates with adverse clinical outcomes. To interrogate its epigenetic function, we engineered isogenic ACSS2‐altered variants via lentiviral transduction in PNET cell lines (BON‐1/QGP‐1), probing its impact on histone acetylation. Western blot showed that overexpressed ACSS2 increased the acetylation modification levels of histones H3 and H4 and key lysine sites (including H3K9, H3K27, and H4K16) at the level of acetylation modification, reflecting the regulation of pan‐acetylation in PNET cells (**Figure** **2**A). In contrast, when ACSS2 was knocked down, the acetylation modification levels of histones H3 and H4 and key lysine sites (including H3K9, H3K27, and H4K16) all exhibited different degrees of impairment compared to controls (Figure 2A; Figure S1C, Supporting Information). ELISA detection of acetyl‐CoA in BON‐1 and QGP‐1 cells showed that ACSS2 overexpression and knockdown resulted in increased and decreased acetyl‐CoA levels, respectively (Figure 2B). Further, we observed that administration of acetate or ACSS2i promoted or depleted acetyl CoA accumulation, which could be attributed to the complementation of the enzymatic reaction by acetate as a substrate and the inhibition of ACSS2 enzymatic activity by ACSS2i (Figure 2C). It was hypothesized that the abnormally increased ACSS2 in the tumor tissues of patients with PNETs may have led to the accumulation of local acetyl‐CoA and increased histone acetylation modification.

Numerous reports have revealed that histone acetylation modifications involved in ACSS2 regulate tumorigenesis and progression, and participate in autophagy, metabolism, drug resistance, and other biological processes.^[^ ^48^, ^49^, ^50^, ^51^ ^]^ To explore the potential regulatory mechanisms of ACSS2 on the biological behavior of PNET cells, we performed four sets of treatments (ACSS2‐OE vs EV, ACSS2‐KO1 vs Ctrl, ACSS2‐KO2 vs DMSO, ACSS2i vs DMSO) and transcriptomics sequencing on the wild‐type BON‐1 cell line. Consistent with the established biological function of ACSS2 as a pivotal acetyl‐CoA synthetase that promotes downstream gene expression via histone acetylation, our analysis specifically focused on genes exhibiting positive correlation with ACSS2 activity and defined by upregulation in ACSS2‐overexpression groups & downregulation in ACSS2‐KO1/KO2 groups & downregulation in ACSS2‐inhibitor‐treated groups.^[^ ^52^, ^53^, ^54^ ^]^ As demonstrated in Figure 2D, this directionality‐aware approach identified 69 consensus genes showing consistent expression patterns across all four comparison pairs.

It is worth noting that genes involved in multiple pathways often serve as hubs that integrate diverse biological signals, such as KRAS and TP53.^[^ ^55^, ^56^, ^57^, ^58^ ^]^ Following this line of thought, we performed pathway enrichment analysis on 69 differentially expressed genes and counted the frequency of their occurrence in the reported gene set. We employed a tripartite prioritization workflow: i) Hub gene identification: Pathway frequency ≥99th percentile vs human genomic baseline (median = 1.2; binomial test *P*<10^−7^);^[^ ^59^ ^]^ ii) Directional consistency: Integration of log_2_FC directionality across all perturbations (Figure S1D, Supporting Information); iii) Biological validation: Benchmarking against oncogenic hubs (KRAS:35 pathways, TP53:22, BRAF:27) (Table S6, Supporting Information).^[^ ^57^ ^]^ And for FASLG (12 pathways), 83% (10/12) were immune‐related (including hsa04210 apoptosis, and hsa04650 NK cytotoxicity), aligning with observed CD8⁺ T cell depletion (Table S7, Supporting Information). This multi‐faceted approach exceeds conventional enrichment analyses by incorporating network centrality.^[^ ^55^ ^]^ The pathway enrichment results showed that the top 14 were ranked according to the frequency of enrichment into pathways, and the gene FASLG was ranked first and enriched in 12 pathways (Figure 2E; Figure S1D and Table S7, Supporting Information). Real‐time quantitative fluorescence PCR (qRT‐PCR) was used to calculate the relative expression of FasL, which was found to increase with elevated expression of ACSS2 and vice versa in BON‐1 and QGP‐1 (Figure 2F). This result further validated the RNA‐seq analysis in vitro at the transcriptional level. Nevertheless, western blot assay of FasL protein expression in BON‐1 and QGP‐1 cells showed unanticipated results. In BON‐1 and QGP‐1 cells, alterations in ACSS2 did not always cause remarkable differences in the amount of cellular FasL protein (Figure S1E, Supporting Information).

Interestingly, reports showed that synthetic mature FasL proteins can be expressed as membrane proteins on the surface of cell membranes (membrane FasL, mFasL) or exocytosed with extracellular (soluble FasL, sFasL). A multi‐institutional validation study on the prediction of high‐risk pancreatic intraductal papillary mucinous neoplasms showed that sFasL was significantly overexpressed in all 60 high‐risk patients.^[^ ^60^ ^]^ Further, abnormally elevated sFasL is a key predictive marker for the identification of patients with autoimmune lymphoproliferative syndrome.^[^ ^61^ ^]^ Based on this, we determined the levels of mFasL and sFasL in BON‐1 and QGP‐1 cells using flow cytometry and ELISA assays, respectively. We found that mFasL expression tended to increase with increased ACSS2 expression (not significant, Figure S1F, Supporting Information), while sFasL exhibited a steady increase in concentration (Figure 2G). Knockdown of ACSS2 or administration of ACSS2i resulted in a decrease in extracellular sFasL concentration, whereas administration of acetate resulted in an increase in extracellular sFasL concentration (Figure 2H). It is hypothesized that in BON‐1 and QGP‐1 cells, ACSS2 may promote FasL transcription, and the increase in FasL protein is mainly manifested by an increase in extracellular sFasL.

### 3.3 Soluble FasL Secretion Induces CD8⁺ T Cell Apoptosis via Fas Signaling

**Figure 3:**
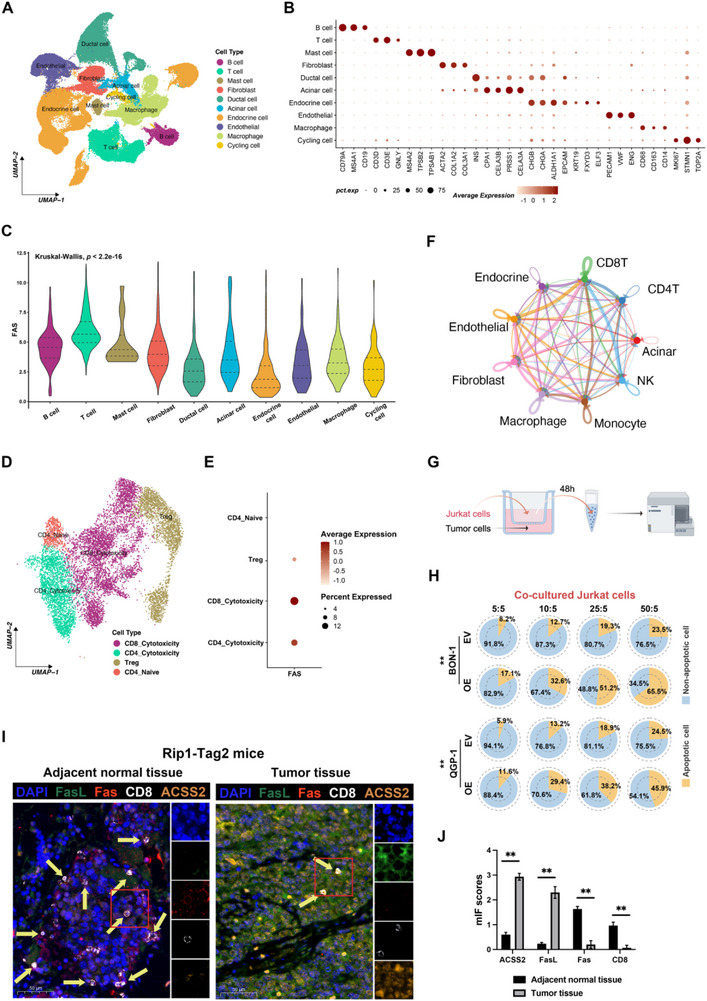
ACSS2‐mediated increase in sFasL in PNET cells induces apoptosis in Jurkat cells through non‐classical manner. A) Dimensionality reduction plot depicting the scRNA‐seq data, identifying a total of 10 major cell types across the tumor tissues. B) Dot plot showing the expression of classical cell type markers across identified cell subpopulations. Dot size represents the percentage of cells expressing each marker and color intensity reflecting average expression. C) Violin plot comparing the expression levels of FAS across major cell subpopulations, with T cells exhibiting significantly higher expression than other subpopulations. D) The uniform manifold approximation and projection (UMAP) representation of four cell subpopulations generated from sub‐clustering T cells. F) Dot plot showing the expression levels of FAS across four T cell subpopulations. G) CellChat infers intercellular communication networks between different cell types. H) The flowchart for the co‐culture of PNETs cells and Jurkat cells. Tumor cells and Jurkat cells were inoculated in the lower and upper chambers of a six‐well plate and co‐cultured for 48 h. Subsequently, Jurkat cells in the upper chamber were aspirated and flow assayed to observe the proportion of cells undergoing apoptosis. Each group of experiments was independently repeated three times. I) Tumor cells with specific ratios (tumor cell: Jurkat cell ratio of 5W:5 W, 10W:5 W, 25W:5 W, and 50W:5 W respectively) wer e co‐cultured with Jurkat cells according to the co‐culture model, and differences in apoptotic proportion of Jurkat cells caused by PNET cells from ACSS2‐OE versus ACSS2‐EV were examined (^**^ *p* < 0.01). J) Multicolor immunofluorescence staining of paraffin‐embedded Rip1‐Tag2 mouse tissue sections for FasL (green), Fas (red), CD8 (white), ACSS2 (orange), and nuclei (DAPI, blue). Yellow arrows indicate magnified cells. To quantify CD8^±^Fas^±^ T cells or ACSS2^±^FasL^±^ tumor cells, 3 randomly photographed spot areas were taken on the 3 slides using a 40 × oil immersion objective, respectively. K) The expression scores of mIF of ACSS2, FASL, Fas, and CD8 in tumor and adjacent normal tissues of Rip1‐Tag2 mice models (^**^ *p* < 0.01, n = 3).

Having established ACSS2's role in FasL induction, we next asked whether this translates to functional immune suppression. Given FasL's apoptotic function,^[^ ^21^ ^]^ we hypothesized that ACSS2‐elevated sFasL depletes CD8⁺ T cells via Fas engagement. Therefore, we examined cells with high expression of Fas receptors in the PNET microenvironment using single‐cell sequencing data (10 × Genomics) from an internal cohort (n = 15) of PNET primary tumor tissues from a pre‐self‐assessment. We identified 10 major cell types and clustered them in the tumor tissue by downscaling the data (**Figure** **3**A; Figure S2A,B, Supporting Information). Dot plots showed the expression of classical cell type markers in the identified cell subpopulations and the subpopulation annotation (Figure 3B; Figure S2C, Supporting Information). The results showed that the expression level of Fas was higher in the T cell subpopulation than in other cell subpopulations (*P* < 0.05, Figure 3C). Since different T cell subtypes exert distinct or even opposite biological functions in tumor development, we further stratified the T cells and clarified Fas expression in representative T cell subpopulations. We found that CD4^+^ and CD8^+^ cytotoxic cells in the subpopulations expressed higher levels of Fas, while Treg cells had lower levels of Fas expression (Figure 3D,E). We re‐clustered CD8^+^ T cells into 7 subpopulations (Clusters 0–6; Figure S3A,B, Supporting Information) and overlaid Fas expression onto the UMAP (Figure S3C, Supporting Information). It is observed that ubiquitous Fas expression in Clusters 0, 1, 2, 4, 5, and 6, with minimal expression in Cluster 3. Further functional enrichment analysis showed Clusters 0, 1, 2, 4, and 5 exhibit enrichment for T cell activation/cytotoxicity pathways (Figure S3D, Supporting Information); Cluster 3 (Fas^low^) shows oxidative metabolism dominance; Cluster 6 had insufficient power for significance due to limited cell numbers (Tables S8 and S9, Supporting Information). Thus, Fas‐mediated apoptosis preferentially depletes tumoricidal CD8^+^ T cell subpopulations, potentially enabling immune escape in PNETs. Furthermore, CellChat analysis suggested that the most prominent cell‐cell communications were observed between CD8^+^ T cells and endocrine cells among the major cellular components^[^ ^62^ ^]^ (Figure 3F; Figure S4A, Supporting Information).

Reports have shown a strong positive correlation between plasma sFasL levels and T‐cell surface Fas/CD95 expression with the propensity of T cells to die.^[^ ^63^ ^]^ Accordingly, we conjectured whether PNETs cells could promote the activation of the local Fas/sFasL system through sFasL expression, which further led to apoptosis of killer T cells with high FasL inflammation in the microenvironment. Since Jurkat cells are commonly used for T cell apoptosis studies, we designed a co‐culture model to verify the above assumptions (Figure 3G).^[^ ^64^, ^65^, ^66^, ^67^, ^68^ ^]^ With different proportions of tumor cells, indirect co‐culture with Jurkat cells showed that the proportion of Jurkat cells undergoing apoptosis gradually increased with the increasing proportion of tumors, and the proportion of Jurkat cells undergoing apoptosis co‐cultured with tumor cells from the ACSS2‐OE group was significantly higher than that from the ACSS2‐EV group (Figure 3H). Further, we found that the addition of ACSS2i reduced the proportion of co‐cultured Jurcat cells undergoing apoptosis in the blank control and ACSS2‐OE groups, and the addition of FasL antibodies had the same effect (Figure S4B, Supporting Information). Further, to validate the single‐cell sequencing analysis and in vitro co‐culture findings, we took tumor tissues from spontaneous PNET mice and adjacent normal pancreatic tissues for sectioning and multi‐color immunofluorescence (mIF) staining (Figure 3I). In normal pancreatic tissues, T cells expressed CD8 (white) and Fas (red) were infiltrated, and most of the mesenchymal‐infiltrating CD8^+^ T cells co‐expressed Fas, which was consistent with our findings in single‐cell sequencing (Figure 3I left and Figure 3J). In contrast, ACSS2 (orange) was highly expressed in PNET, the interstitium was diffused with abundant FasL (green), and CD8^+^ T cells were impaired, resulting in an intuitively reduced cell percentage (Figure 3I right and Figure 3J). The proportion of infiltrating CD8^+^ T cells in them was much lower than that in adjacent normal tissues. In conclusion, these findings suggest that ACSS2 may promote the activation of the Fas/sFasL system through the expression of sFasL, which further leads to apoptosis of T cells with high FasL expression locally in the tumor.

### 3.4 Transcription‐Promoting Factor AATF Synergizes with ACSS2 to Increase FasL Expression

**Figure 4:**
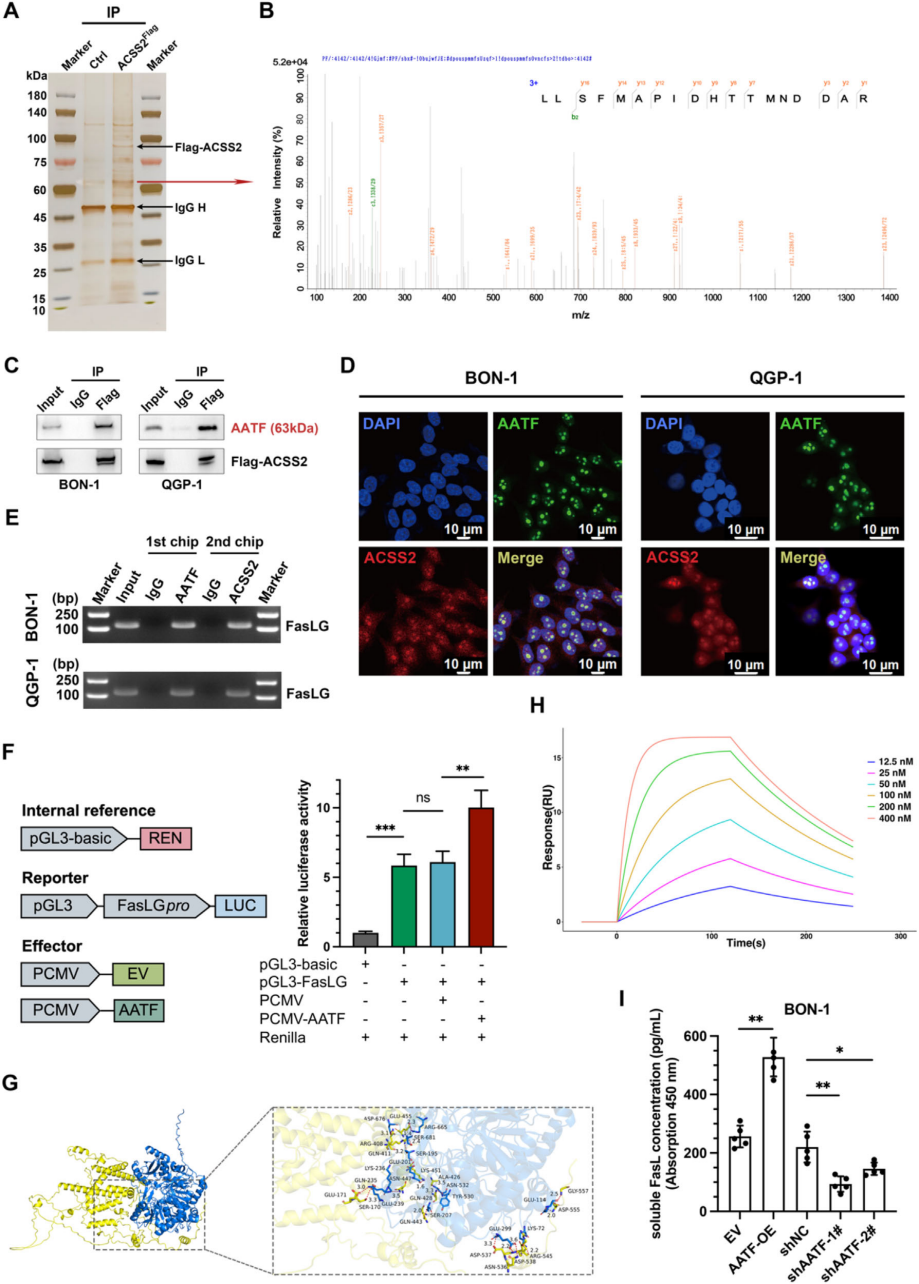
Transcription‐promoting factor AATF synergizes with ACSS2 to increase FasL expression A) Silver staining of SDS‐PAGE gels indicated ACSS2‐interacting proteins. Flag‐ACSS2 expression stable BON‐1 cells were lysed and immunoprecipitated with anti‐Flag Ab or rabbit IgG control Ab, and then subjected to SDS‐PAGE gel and silver staining. B) Representative tandem MS spectrum of the peptide LLSFMAPIDHTTMNDDAR from AATF as determined by IP‐Mass Spec. C) AATF was further identified to bind with ACSS2 by immunoprecipitation. Flag‐ACSS2 expression stable BON‐1 cells were lysed and immunoprecipitation with anti‐Flag Ab or abbit IgG control Ab, and then subjected to immunoblotting using antibodies against AATF. D) Endogenous ACSS2 colocalized with AATF in PNET cells. Localization of AATF (green) and ACSS2 (red) in BON‐1 and QGP‐1 cells was detected by double immunofluorescence labeling and confocal microscopy. The merged image with the yellow signal represented their colocalization. E) A ChIP‐re‐ChIP assay was conducted using anti‐AATF antibody first (AATF) in BON‐1 and QGP‐1 cells. The eluents were then subjected to a second ChIP assay using anti‐Flag‐ACSS2 antibody (AATF + Flag‐ACSS2) or control IgG antibody (AATF + IgG) (n = 3). F) Dual luciferase validation of AATF positive regulation of FasL transcription. Schematic representations of the reporter and effector constructs used in the dual‐LUC assay. Firefly luciferase (LUC) driven by the *FasLG* promoter was used as the reporter. Renilla luciferase (REN) was used as an internal control. G) The binding sites of ACSS2 and AATF protein were simulated using AutoDock Vina v.1.2.2 molecular docking analysis, whose low binding energy is −11.3 kcal mol^−1^. H) Surface plasmon resonance (SPR) analysis using a Biacore 8K system (Cytiva) quantified the binding kinetics and affinity between the analyte (AATF) and immobilized ligand (ACSS2). Six serially diluted concentrations of AATF (range: 12.5 – 400 nm) were injected over ACSS2‐coupled CM5 sensor chips. Real‐time binding curves demonstrated concentration‐dependent responses, yielding an equilibrium dissociation constant (K*_D_*) of 33.3 ± 1.7 nm, indicative of high‐affinity molecular recognition. I) Overexpression (or knockdown) of AATF in PNET cells could lead to increased (or decreased) sFasL levels. ELISA assay of sFasL concentration levels in BON‐1 cell culture supernatants from different treatment groups. Five independent repetitions for each group (^*^ *p* < 0.05, ^**^ *p* < 0.01).

To explore the mechanism of transcriptional regulation of FasL by ACSS2, we performed an MS assay on the BON‐1 cell line (ACSS2^Flag^ vs EV) based on the results of the silver staining assay (**Figure** **4**A,B). Combining the results of silver staining and MS assay, we speculated that there might be potential binding between ACSS2 and the apoptosis‐antagonizing transcription factor (AATF, also named Che‐1) from 42 identified proteins (Table S10, Supporting Information). Since literature reports that AATF is now recognized as a multifunctional protein involved in cell cycle regulation, apoptosis inhibition, stress response, and cancer progression.^[^ ^69^, ^70^, ^71^ ^]^ We speculated whether AATF is involved in the transcriptional regulation of ACSS2 on FasL in PNET cells. As expected, the results of the Co‐IP assay and immunofluorescence assay showed that ACSS2 might bind to AATF and spatially co‐localize in BON‐1 and QGP‐1 cells, which further confirmed this speculation (Figure 4C,D). We further designed in vitro experiments to investigate whether AATF is involved in the transcriptional promotion of ACSS2 on FasL. A ChIP‐re‐ChIP assay was performed to test the co‐occupancy of AATF and ACSS2 on the *FasLG* promoter. The results demonstrated that the promoter region of *FasLG* was occupied from the DNA recovered from the immunoprecipitation complex using specific antibodies for AATF and ACSS2 but not the control IgG (Figure 4E). Subsequent dual luciferase reporter gene analysis confirmed that AATF exerts a positive pro‐transcriptional regulatory effect on FasL. (Figure 4F). Molecular docking and dynamics simulations explored the stability of the ACSS2‐AATF interaction. A 3D binding model showed specific amino acid interactions contributing to structural stability (Figure 4G). SPR‐based binding assays (Biacore 8K) revealed a high‐affinity interaction between ACSS2 and AATF, with a K*_D_* of 33.3 ± 1.7 nm (Figure 4H). Concentration‐dependent sensorgrams confirmed rapid association and stable complex formation, supporting biologically relevant complex formation. Overexpression or knockdown of AATF resulted in increased or decreased FasL transcript levels (Figure S5A, Supporting Information) and sFasL extracellular concentration (Figure 4I; Figure S5B, Supporting Information). These data suggest the potential role of AATF in regulating ACSS2‐mediated acetyl‐CoA metabolism and epigenetic reprogramming in neoplasia.

### 3.5 AATF acts as an Intranuclear Guide that Binds ACSS2 and Localizes to the FasL Promoter Region to Enhance FasL Transcription in Concert with Histone Pan‐Acetylation Modification

**Figure 5:**
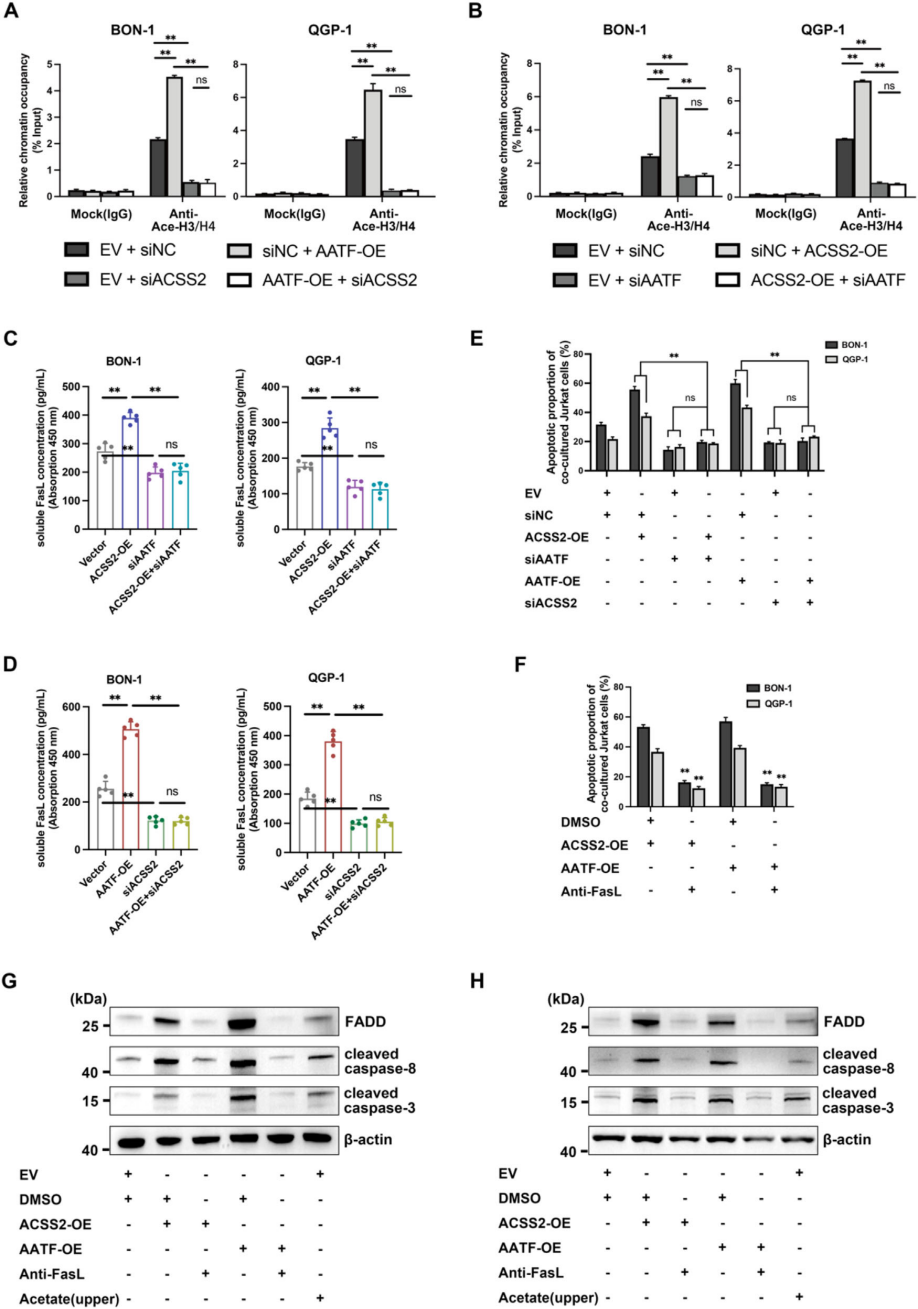
AATF acts as an intranuclear guide that binds ACSS2 and localizes to the *FasLG* promoter region to enhance transcription in concert with histone pan‐acetylation modification. A) AATF binding to the *FasLG* promoter region and promoting transcription requires synergistic ACSS2‐mediated histone acetylation modifications. ChIP qRT‐PCR detection of AATF occupancy on the promoters of *FasLG* in BON‐1 cells and QGP‐1 cells. ChIP‐level antibodies against acetylation‐modified H3 and H4 were used to specifically pull down H3ac/H4ac histones in each treatment group. Silencing of ACSS2 expression after overexpression of AATF reversed the increase in *FasLG* promoter region occupancy caused by AATF overexpression (siNC + AATF‐OE vs EV + siNC, siNC + AATF‐OE vs AATF‐OE + siACSS2). The decrease in *FasLG* promoter region occupancy after silencing ACSS2 expression was not reversed by overexpression of AATF (EV + siACSS2 vs EV + siNC, EV + siACSS2 vs AATF‐OE + siACSS2) (^**^ *p* < 0.01, ns indicates not significant). B) Enhancement of *FasLG* transcription by ACSS2 requires endogenous guidance of AATF and pro‐transcriptional regulation of the *FasLG* promoter region by AATF. ChIP qRT‐PCR detection of AATF occupancy on the promoters of *FasLG* in BON‐1 cells and QGP‐1 cells. ChIP‐level antibodies against acetylation‐modified H3 and H4 were used to specifically pull down H3ac/H4ac histones in each treatment group. Silencing of AATF expression after overexpression of ACSS2 reversed the increase in *FasLG* promoter region occupancy caused by ACSS2 overexpression (siNC + ACSS2‐OE vs EV + siNC, siNC + ACSS2‐OE vs ACSS2‐OE + siAATF). The decrease in *FasLG* promoter region occupancy after silencing AATF expression was not reversed by overexpression of ACSS2 (EV + siAATF vs EV + siNC, EV + siAATF vs ACSS2‐OE + siAATF) (^**^ *p* < 0.01, ns indicates not significant). C,D) ELISA assay of sFasL concentration levels in BON‐1 and QGP‐1 cell culture supernatants from different treatment groups. Consistency in intergroup comparisons of different cell lines. Five independent repetitions for each group (^**^ *p* < 0.01, ns indicates not significant). E) The percentage of apoptotic Jurkat cells after co‐cultured with BON‐1 and QGP‐1 cells among distinct groups (^**^ *p* < 0.01, ns indicates not significant). F) FasL antibody (0.1 µg mL^−1^) was detected after 48 h of incubation in the lower chamber of co‐cultured six‐well plates (^**^ *p* < 0.01). G,H) Validation of expression levels of key pro‐apoptotic proteins in the Fas/FasL pathway. After overexpression of ACSS2 or AATF, immunoblotting was performed using FADD, cleaved caspase‐8, and cleaved caspase‐3 antibodies with β‐actin as upload control in BON‐1 (G) and QGP‐1 (H) cell lines, respectively.

To explore the potential relationship between AATF and ACSS2 in the regulation of expression of FasL, we performed a ChIP‐qPCR assay using by pan‐acetylated antibody. The results showed that when independently overexpressing AATF or knocking down ACSS2, the relativistic chromatin occupancy of FasL exhibited a significant elevation (siNC + AATF‐OE vs EV + siNC) or decreased (EV + siACSS2 vs EV + siNC) (**Figure** **5**A). However, when overexpression of AATF was combined with knockdown of ACSS2, it only showed a similar modulatory effect as knockdown of ACSS2 alone, but was not rescued by simultaneous overexpression of AATF (Figure 5A). Correspondingly, we observed a similar phenomenon when overexpressing ACSS2 combined with the knockdown of AATF (Figure 5B). The above findings tentatively suggest that the transcriptional regulation of FasL by AATF is dependent on increased chromatin accessibility (caused by ACSS2‐mediated increase in histone acetylation modification), whereas the transcriptional regulation of FasL by ACSS2 is dependent on the direct regulation of the FasL promoter by the transcription‐promoting factor AATF. As evidence, we further assayed sFasL concentration using an ELISA detection kit to validate the above group comparison results (Figure 5C,D). The data showed that ELISA results consistent with the ChIP‐qPCR assay appeared in the expression of sFasL in the corresponding groups, both in BON‐1 and QGP‐1 cells. Meanwhile, the proportion of Jurkat cells undergoing apoptosis was examined by a co‐culture model, and the results confirmed that the overexpression of ACSS2 combined with the knockdown of AATF exhibited a similar proportion of apoptosis as the knockdown of AATF alone. Correspondingly, the overexpression of AATF combined with knockdown of ACSS2 exhibited a similar proportion of apoptosis as knockdown of ACSS2 alone (Figure 5E). The effect of increased apoptosis in Jurkat cells induced by overexpression of ACSS2 or AATF could be impaired by FasL antibodies (Figure 5F). Therefore, ACSS2 non‐redundant binds AATF to synergistically modulate sFasL expression through improving histone pan‐acetylation modification.

### 3.6 Targeting ACSS2/AATF‐sFasL Axis Abrogates CD8⁺ T Cell Apoptosis and Restores Anti‐Tumor Immunity in PNET Patient‐Derived Models

The Fas/FasL pathway has been reported to form a death‐inducing signaling complex through the binding of signaling molecules to the death structural domain of the receptor, which in turn activates downstream members such as caspase‐8 and caspase‐3, leading to apoptosis.^[^ ^72^ ^]^ Upon overexpression of ACSS2 or AATF, respectively, elevated caspase‐8 and caspase‐3 enzyme activities were observed in Jurkat cells after co‐culture with BON‐1 or QGP‐1 compared to controls, and addition of FasL antibody reversed the elevation of cleaved caspase‐8 (Figure S6A, Supporting Information) and cleaved caspase‐3 activities (Figure S6B, Supporting Information). Since acetate has the ability to permeate through the filter membrane of the co‐culture chambers, we observed the changes in caspase enzyme activity following the administration of acetate to the upper and lower chambers, respectively. The results showed that the addition of acetate to both the upper and lower chambers, respectively, resulted in elevated caspase‐8 (Figure S6A, Supporting Information) and caspase‐3 enzyme activities (Figure S6B, Supporting Information) in co‐cultured Jurkat cells compared to the control group. The above results suggest that the enhanced sFasL by ACSS2 in conjunction with AATF exerts a pro‐apoptotic effect by activating intracellular caspase‐8 and caspase‐3 enzymes. As observed, western blot experiments in BON‐1 (Figure 5G) and QGP‐1 (Figure 5H) cells showed that overexpression of ACSS2 or AATF increased the expression of FADD, a key pro‐apoptotic molecule in the Fas/FasL pathway, and further activated the expression of cleaved caspase‐8 and cleaved caspase‐3. The addition of acetate to the upper chamber achieved similar bonds. FasL antibodies, on the other hand, attenuated the elevation of pro‐apoptotic‐related proteins caused by overexpression of ACSS2 or AATF (Figure 5G,H).

**Figure 6:**
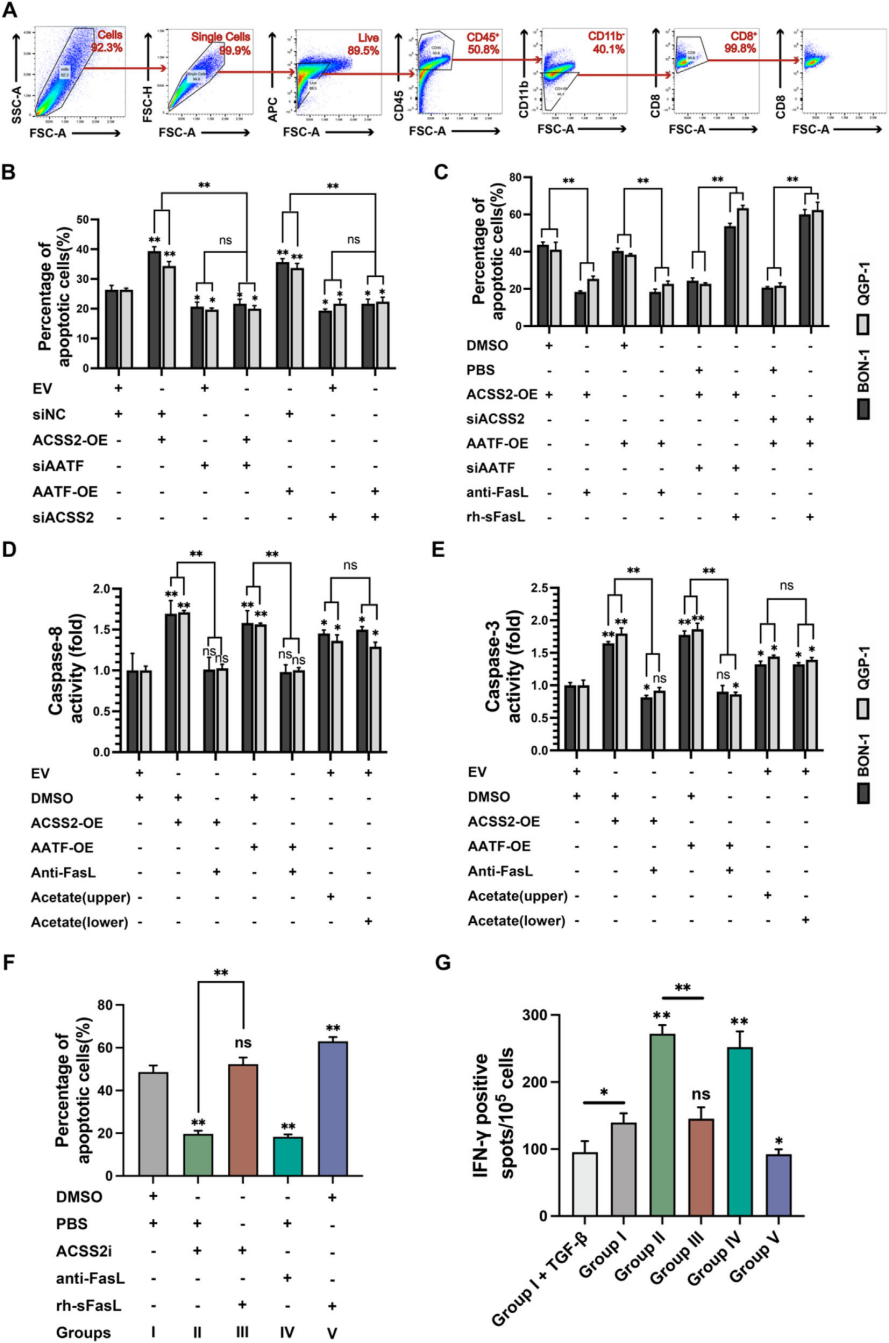
Detection of apoptotic proteases in primary T cells and histological validation of the correlation between ACSS2 and FasL expression in PNETs. A) Flow sorting procedure for primary CD8^+^ T cells. B,C) The percentage of apoptotic primary CD8^+^ T cells after co‐cultured with BON‐1 and QGP‐1 cells among distinct groups. Animal‐free recombinant human soluble Fas ligand (rh‐sFasL, 4 units/mL) was detected after 18 h co‐incubation of independent treatment (^*^ *p* < 0.05, ^**^ *p* < 0.01, ns indicates not significant). D,E) Changes in the activity of caspase‐8 (D) and caspase‐3 (E) in BON‐1 and QGP‐1 cell lines were determined by fluorescence assay after the corresponding treatments. Acetate (1 mm) was detected after 48 h of independent treatment in the upper and lower chambers of co‐cultured six‐well plates. FasL antibody (0.1 µg mL^−1^) was detected after 48 h of incubation in the lower chamber of co‐cultured six‐well plates (^*^ *p* < 0.05, ^**^ *p* < 0.01, ns indicates not significant). F) The percentage of apoptotic primary CD8^+^ T cells after co‐cultured with PNET patient‐derived organoids (PDOs) among distinct groups. The ACSS2i (0.5 µM), anti‐FasL antibody (0.1 µg mL^−1^), or animal‐free recombinant human soluble Fas ligand (rh‐sFasL, 4 units/mL) was added to one well in a six‐well plate and then independently treated and co‐incubated for 48 h (^**^ *p* < 0.01, ns indicates not significant). G) Bar plot showing the IFN‐γ positive spots/10^5^ cells in the different suppression and co‐culturing conditions as indicated in the plot. TGF‐β treatment was added as a negative control for CD8^+^ T cell suppression. Error bars indicate mean ± SEM (^*^ *p* < 0.05, ^**^ *p* < 0.01, ns indicates not significant).

Further, we successfully sorted and cultured primary CD8^+^ T cells from 3 PNET patients and elucidated the apoptotic ratio via the co‐culture model (**Figure** **6**A; Table S11, Supporting Information). We found that overexpression of ACSS2 or AATF resulted in increased apoptosis of primary CD8^+^ T cells co‐cultured with PNET cells, while knockdown of ACSS2 or AATF resulted in decreased apoptosis (Figure 6B). Further, overexpression of ACSS2 while knocking down AATF (or overexpression of AATF while knocking down ACSS2) resulted in a stable reduction in the proportion of apoptotic primary CD8^+^ T cells (Figure 6B). Additionally, knockdown of ACSS2 (or AATF) with or without overexpression of AATF (or ACSS2) did not cause significant changes in apoptosis, both in BON‐1 and QGP‐1 cells (Figure 6B). Co‐incubation of FasL antibodies counteracted the increase in apoptosis of primary CD8^+^ T cells induced by overexpression of ACSS2 or AATF, whereas co‐incubation of rh‐sFasL proteins rescued the decrease in apoptosis induced by knockdown of ACSS2 or AATF (Figure 6C). Correspondingly, the trends of altered caspase‐8 and caspase‐3 enzyme activities were validated in vitro in primary CD8^+^ T cells of patient origin (Figure 6D,E). Elevated caspase‐8 and caspase‐3 enzyme activities were observed in primary CD8^+^ T cells co‐cultured with PNET cells overexpressing ACSS2 or AATF compared to controls. Co‐incubation with FasL antibodies reversed the elevation of caspase‐8 and caspase‐3 enzyme activities caused by overexpression of ACSS2 or AATF (Figure 6D,E). Meanwhile, the addition of acetate to the upper or lower chambers resulted in increased caspase‐8 and caspase‐3 enzyme activities in primary CD8^+^ T cells co‐cultured compared with the control group (EV + DMSO), respectively (Figure 6D,E).

To evaluate the therapeutic potential of targeting the ACSS2/AATF‐sFasL axis, we employed PNET patient‐derived organoids (PDOs) from three independent donors. PDO‐CD8⁺ T‐cell co‐cultures showed that ACSS2i or anti‐FasL reduced apoptosis; rh‐sFasL enhanced it. Rescue experiments confirmed sFasL as the key ACSS2 effector (Figure 6F; Figure S6C, Supporting Information). Co‐incubation of PDOs with ACSS2i or anti‐FasL antibodies demonstrates the advantage of enhancing the cytotoxic CD8^+^ T cells activity (Figure 6G). While the addition of rh‐sFasL caused more inhibition of the cytotoxic activity of CD8^+^ T cells than did the blank group (Group V vs I). It is notable that the rh‐sFasL reversed the increase of cytotoxic CD8^+^ T cell activity caused by ACSS2i, which conforms to the previous observations (Figure 6F,G).

### 3.7 Histological Expression Correlation Statistics of a Clinical In‐House Cohort and In Vivo Validation of Spontaneous Tumor Mouse Models

**Figure 7:**
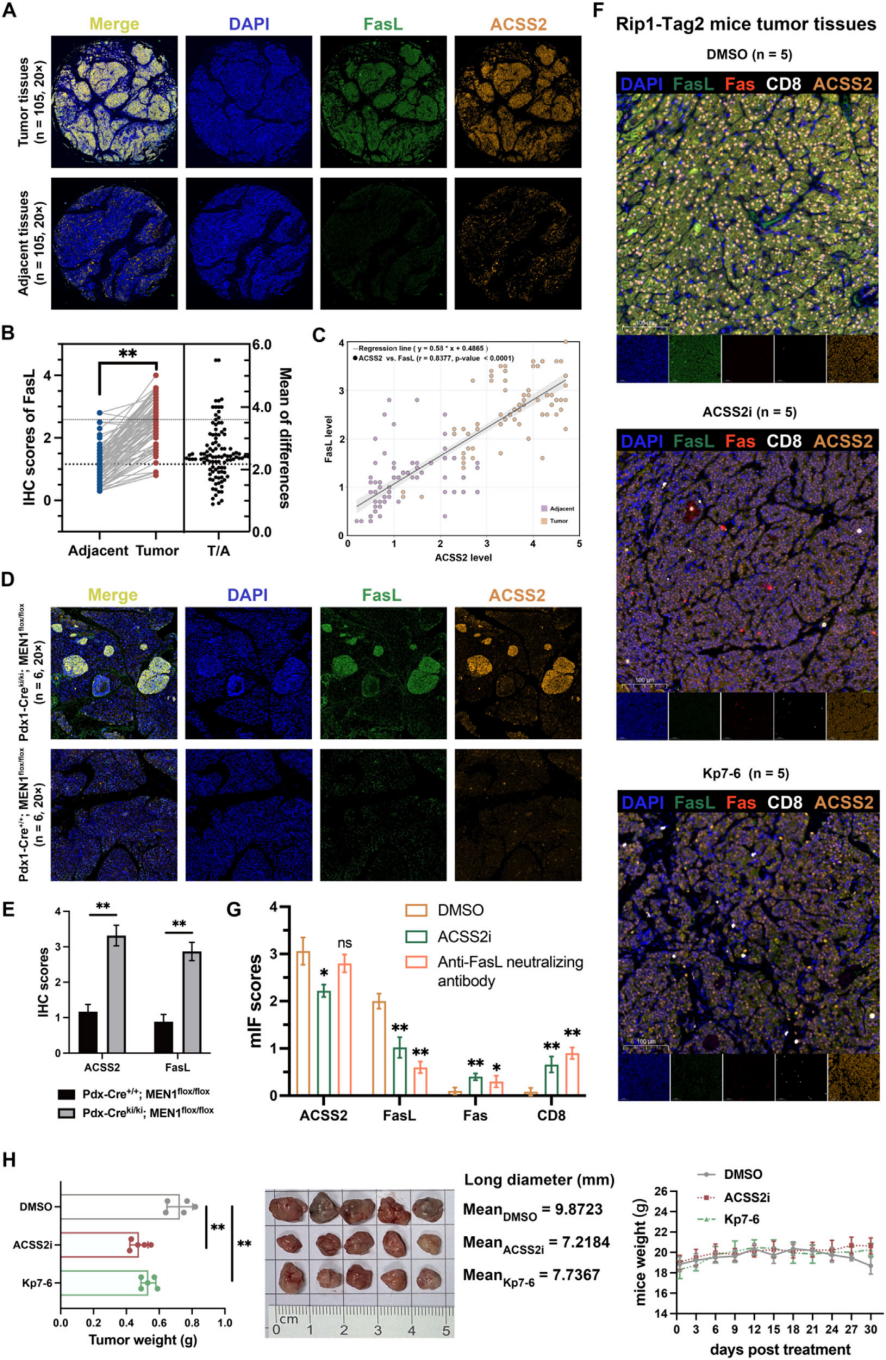
Histological expression correlation statistics and in vivo validation of mouse models of spontaneous tumors. A) Multiplex immunohistochemical (mIHC) staining of human PNET tissues and paired adjacent normal tissues arrays using ACSS2 and FasL‐specific antibodies (n = 105). B) FasL expression was significantly higher in human PNET tissues than in paired adjacent normal tissues in IHC staining of tissue arrays (^**^ *p* < 0.01, n = 105). C) The expression level of FasL was positively correlated with the expression of ACSS2. Each data point represents one patient. The higher the expression of ACSS2, the higher the expression level of FasL. D) The mIHC staining of tumor tissues from mice models of spontaneous PNET and paired adjacent normal tissues using ACSS2 and FasL‐specific antibodies (n = 6). E) ACSS2 and FasL expression were significantly higher in tumor tissues from mice models of spontaneous PNET than in paired adjacent normal tissues in IHC staining (^**^ *p* < 0.01, n = 6). F) The mIF staining of paraffin‐embedded Rip1‐Tag2 mouse tissue sections for FasL (green), Fas (red), CD8 (white), ACSS2 (orange) and nuclei (DAPI, blue). To quantify CD8^±^Fas^±^ T cells or ACSS2^±^FasL^±^ tumor cells, 3 randomly photographed spot areas were taken on the 3 slides using a 40 × oil immersion objective, respectively. G) The mIF scores of ACSS2, FASL, Fas, and CD8 in tumor tissues from control and drug‐treated groups of Rip1‐Tag2 mice models (^*^ *p* < 0.05, ^**^ *p* < 0.01, n = 5). H) Tumor tissues of Rip1‐Tag2 mice were collected and photographed (scale bar = 1 cm). Tumor size and weight were measured on the sacrificed day (mean ± SD, n = 5, ^**^ *p* < 0.01).

To investigate the expression of FasL in clinical patients and the relationship between ACSS2 and FasL expression, our mIHC assay of tissues from 105 patients with paired PNETs showed that the expression of FasL in tumor tissues was higher than that in adjacent normal tissues (**Figure** **7**A,B), and there was a strong positive correlation with the expression of ACSS2 (Figure 7C). In the genetic background of primary PNET patients, MEN1 is a commonly mutated gene, and most of them are germline deletion mutations.^[^ ^73^ ^]^ We constructed a *Men1*‐deficient gene‐edited mice model of PNET spontaneous tumor, recapitulating human *Men1*‐associated PNETs.^[^ ^29^, ^74^ ^]^ In this study, we performed mIHC comparisons utilizing pancreatic tissues from six genetically engineered mice with wild‐type mice, and the results showed that the primary tumors were found to be highly enriched in clear ACSS2 and FasL proteins (Figure 7D,E). The results suggested that apoptosis of CD8^+^ T cells originally co‐expressing Fas might have occurred due to the continuous expression of ACSS2 and FasL in tumor cells.

**Figure 8:**
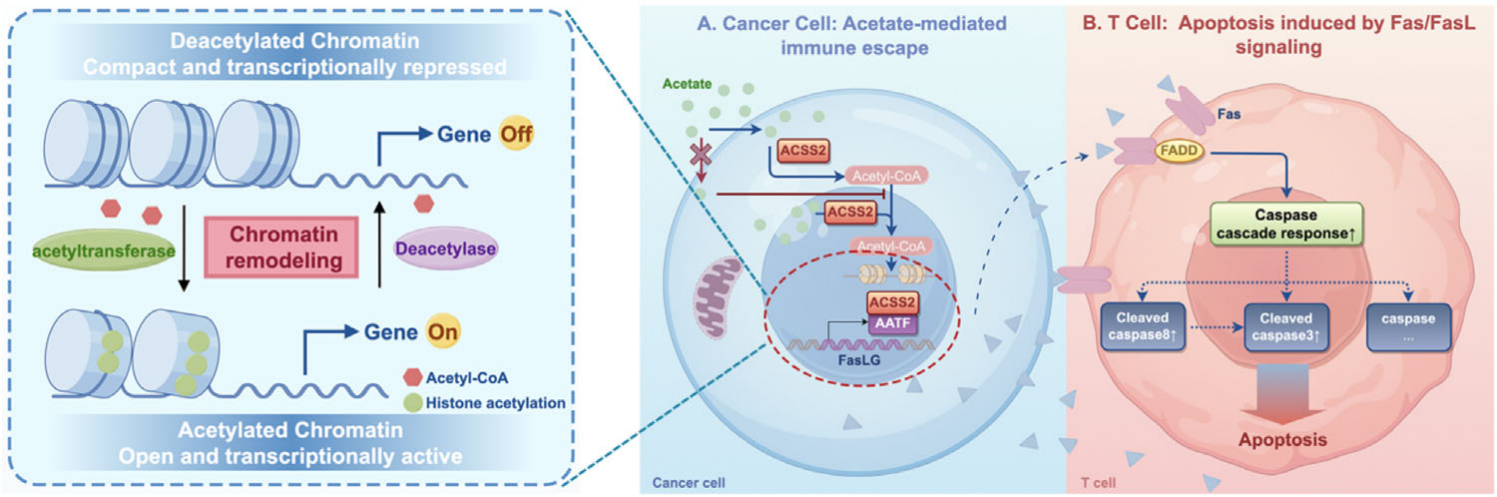
The proposed working model of this study.

Further, we constructed and validated the Rip1‐Tag2 transgenic mouse model (Figure S7A,B and Table S12, Supporting Information). Local FasL expression in pancreatic tumors of Rip1‐Tag2 mice was reduced, and CD8^+^Fas^+^ T cell infiltration was increased after application of ACSS2 inhibitor (Figure 7F,G). Original images of the Rip1‐Tag2 mouse tumor and quantified the tumor size, and provided the tumor growth tracking data as shown in Figure 7H and Figure S7C (Supporting Information). Manders’ overlap coefficient (MOC) quantified that ACSS2 and sFasL exhibit compartmentalized co‐localization specifically in tumor epithelial cells (Table S13, Supporting Information). As a Fas‐mimetic peptide, Kp7‐6 is a Fas/FasL antagonist that protects mouse cells from Fas‐mediated apoptosis.^[^ ^75^, ^76^ ^]^ Our application of Kp7‐6 treatment to Rip1‐Tag2 mice revealed a significant reduction of FasL and an increase in CD8^+^Fas^+^ T‐cell infiltration in the local infiltration of tumors without altering ACSS2 expression. Taken together, this finding reveals that the ACSS2/AATF/FasL axis could trigger the apoptosis of T cells via histone pan‐acetylation modification (**Figure** **8**).

## 4 Discussions

Protein post‐translational modification is a key link in protein expression, in which acetylation modification is associated with diseases such as tumors, aging, and infection, and is involved in biological processes such as DNA damage repair, protein folding, metabolism, and drug resistance.^[^ ^51^, ^77^ ^]^ With the identification of histone acetyltransferases, histone deacetylases, and acetylated lysine‐binding proteins as transcriptional regulators, the mystery of histone acetylation modifications in transcriptional regulation is gradually being revealed.^[^ ^78^ ^]^ ACSS2, as a key member of acetyl‐CoA synthetase, has been found to have an important role in tumor metabolism and progression in recent years. In addition, as one of the acetyl‐Coenzyme A synthases, ACSS2 could promote acetyl‐CoA anabolism using acetate as a substrate. Reports have shown that ACSS2 acts as a lactose coenzyme A synthase in combination with KAT2A as a lactosyltransferase for histone lactosylation and tumor immune evasion.^[^ ^79^ ^]^ Herein, this study focuses on the function of ACSS2 in PNETs and reveals a novel mechanism by which it affects tumor biological behavior through histone acetylation modification. Beyond its canonical metabolic functions, our data reveal ACSS2‐synthesized acetyl‐CoA as an epigenetic orchestrator of immune evasion via histone hyperacetylation.

The Fas/FasL pathway occupies a central position in the regulation of apoptosis, and it is involved in several biological processes such as tumor immune escape, maintenance of tissue homeostasis, and pathogen clearance by triggering death receptor‐mediated apoptosis.^[^ ^80^, ^81^ ^]^ In addition, the Fas/FasL pathway binds to signaling molecules through binding and the death structural domains of its receptor to form a death‐inducing signaling complex, which in turn activates the downstream caspase enzyme cascade reaction and ultimately causes apoptosis.^[^ ^82^ ^]^ Among them, caspase‐8, as the first key performer after the activation of the Fas/FasL pathway, is rapidly activated upon receiving the upstream signal, which in turn cleaves and activates downstream caspase‐3 and other members.^[^ ^83^ ^]^ caspase‐3, as a key effector enzyme in the process of apoptosis, is responsible for executing the final apoptotic steps such as DNA breakage and cytoskeleton degradation.^[^ ^84^, ^85^, ^86^ ^]^ Taken together, caspase‐8 and caspase‐3 play indispensable roles in apoptosis mediated by the Fas/FasL pathway, and are key bridges connecting Fas/FasL signaling to the execution steps of apoptosis. In tumor immunity, the Fas/FasL pathway also plays a central role, not only in mediating off‐target “bystander” killing of antigen‐negative tumor cells, but also in the immune escape mechanism from tumors.^[^ ^24^ ^]^ Therefore, the study of the Fas/FasL pathway contributes to an in‐depth understanding of the regulatory mechanism of apoptosis and provides new ideas for tumor therapy. In this study, we observed that ACSS2 not only promoted the transcription of FasL but also affected its expression at the protein level. This finding reveals that ACSS2 may be involved in the immune escape mechanism of tumors by regulating the Fas/FasL pathway and affecting T cell apoptosis in the microenvironment of PNETs. Notably, we found a non‐redundant synergistic interaction between ACSS2 and the transcription factor AATF to co‐regulate FasL expression. This synergistic effect not only enhanced the transcriptional level of FasL but also promoted its expression at the protein level, further supporting the important role of ACSS2 in the regulation of the Fas/FasL pathway.

Apoptosis antagonizing transcription factor (AATF, also named Che‐1), the encoding gene is located on human chromosome 17q12, and the gene product contains a leucine zipper, which is a characteristic motif of the transcription factor, in addition to a nuclear localization signal (NLS) sequence and a phosphorylation site (ser166), and these functional regions play an important role in the regulation of AATF activity and function.^[^ ^69^, ^87^ ^]^ In cells, AATF is mainly located in the nucleus and is targeted intranuclear via NLS sequences. In addition, AATF interacts with a variety of transcription factors to regulate the expression of several metabolism‐related genes and influence the metabolic activity of cells.^[^ ^70^, ^71^ ^]^ Therefore, an in‐depth study of the function and regulatory mechanisms of AATF is important for understanding cell biological processes as well as the occurrence and development of diseases. In this study, we found that ACSS2 and AATF have tight synergistic roles in regulating the expression of FasL. ACSS2 provides substrates for histone acetylation by catalyzing the synthesis of acetyl coenzyme A, thereby increasing chromatin accessibility and promoting gene transcription. AATF, on the other hand, acts as a transcriptional activator, directly binding to the promoter region of FasL and regulating its transcriptional activity. Both are indispensable in the regulation of FasL expression. The acetylation modification of ACSS2 provides a favorable chromatin environment for AATF binding, while AATF binding further enhances the transcriptional activity of FasL. This synergistic effect makes ACSS2 and AATF exhibit potent effects in regulating FasL expression.

Our finding that ACSS2/AATF epigenetically licenses FasL expression provides a mechanistic rationale for targeting this axis. Notably, ACSS2 inhibitors not only reduce acetyl‐CoA but also reverse histone hyperacetylation at FasLG loci, explaining their efficacy in rescuing CD8⁺ T cell infiltration. However, this study also has some limitations. First, our study focused on in vitro experiments and animal models, and although these results support our hypothesis, further validation in clinical samples is needed. Second, although we found that the synergistic interaction between ACSS2 and AATF plays an important role in regulating FasL expression, the specific molecular mechanisms need to be further investigated in depth, including the possible mechanism of whether ACSS2 or AATF can promote the exocytosis of mature FasL proteins. While our transgenic models and primary co‐cultures provide mechanistic insights, the absence of immune‐intact PNET xenografts limits direct evaluation of tumor‐immune dynamics in vivo. Future studies will employ humanized NSG mice implanted with patient‐derived tumors to assess ACSS2/AATF targeting in clinically relevant microenvironments.

Collectively, the present study revealed the posttranslational control of ACSS2 in PNETs via the acetylation and its mechanism of inducing apoptosis of T cells in the microenvironment of PNETs via the Fas/FasL pathway and then promoting tumor immune escape. Our findings not only deepen the understanding of the biological behavior of PNETs but also provide a theoretical basis for the development of targeted therapeutics against ACSS2. In the future, we plan to validate ACSS2 expression and its relationship with AATF and FasL in more clinical samples to further confirm our findings. In addition, we will also conduct in‐depth studies on the molecular mechanisms by which ACSS2 and AATF synergistically regulate the expression of FasL and the potential mechanisms that may be involved in the exocytosis of mature FasL proteins, with a view to finding more specific therapeutic targets. Through these studies, we expect to be able to provide more effective therapeutic options for patients with PNETs and improve their prognosis.

## Conflict of Interest

The authors declare no conflict of interest.

## Author Contributions

Q.D., T.W., and Y.W. contributed equally to this work. X.J.Y., X.W.X., Q.S.H., and Y.Q. performed in conceptualization. Q.D., B.R.L., Z.Y., S.R.J., and Y.Q. performed in methodology. Q.D., T.W., Z.Y., X.P., Y.W., G.X.F., D.S.J., and Y.Q. performed in the investigation. Q.D., T.W., J.F.X., and Z.L. performed in visualization. G.X.F., S.R.J., and Y.Q. performed in supervision. Q.D., T.W., Y.W., and X.P. performed in writing.

## Supporting information



Supporting Information

Supporting Information

## Data Availability

All data associated with this study are present in the paper or the Supplementary Materials. Single cell RNA‐seq data was uploaded to GEO database (GSE256136). Any additional information required to reanalyze the data reported in this work paper is available from the lead contact upon request.
