# Supplementary material for: ACSS2/AATF Drives Soluble FasL‐Mediated CD8+ T Cell Apoptosis in Pancreatic Neuroendocrine Tumors
Source: Adv Sci (Weinh). 2025 Aug 12;12(40):e06883. doi: 10.1002/advs.202506883 (PMC12561415; doi:10.1002/advs.202506883)
Supplement: Supplementary file 1 — Supporting Information [file ADVS-12-e06883-s002.docx]

**Supplementary Figure 1**


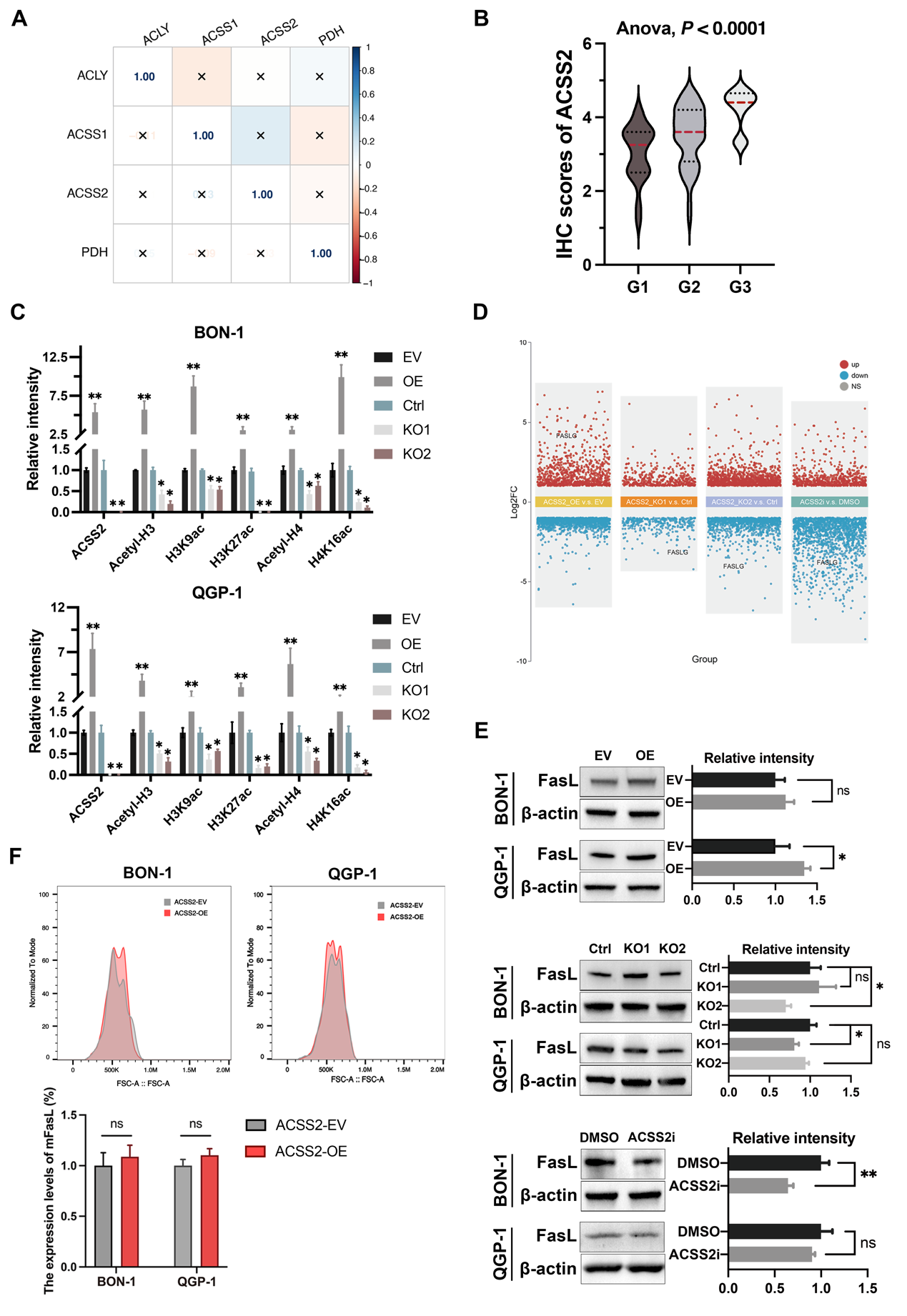


**Supplementary figure 1. Expression correlation analysis of four acetyl-CoA synthetases and quantification of acetylation level and membrane FasL.**

1. Expression correlation analysis and the coefficients between ACLY, ACSS1, ACSS2 and PDH (“**×**” indicates not significant).
2. Correlation between ACSS2 expression levels and grades of a clinical in-house cohort in Fudan University Shanghai Cancer Center (FUSCC, n = 105).
3. The corresponding quantitative values of the immunoblot bands shown in Fig. 2A. The calculate was conducted with three independent biological replicates.
4. Differential expression volcano plot of the *FasLG* gene in four comparison pairs in BON-1 cell line.
5. Immunoblotting using FasL antibody after overexpression of ACSS2, knockdown of ACSS2, or administration of ACSS2 inhibitor, with β-actin as the upload control. The corresponding quantitative values of protein expression were shown to the right of the immunoblot bands.
6. Flow cytometry was performed to detect the level of FasL expression on the membrane surface of BON-1 and QGP-1 cells after ACSS2 overexpression.

Data information: Each value represents mean ± SD. The difference between two groups was calculated using Student’s *t* test. One-way ANOVA analysis with Dunnett’s test was used for multiple comparisons. **P* < 0.05, ** *P* < 0.01, ns indicates not significant.

**Supplementary Figure 2**


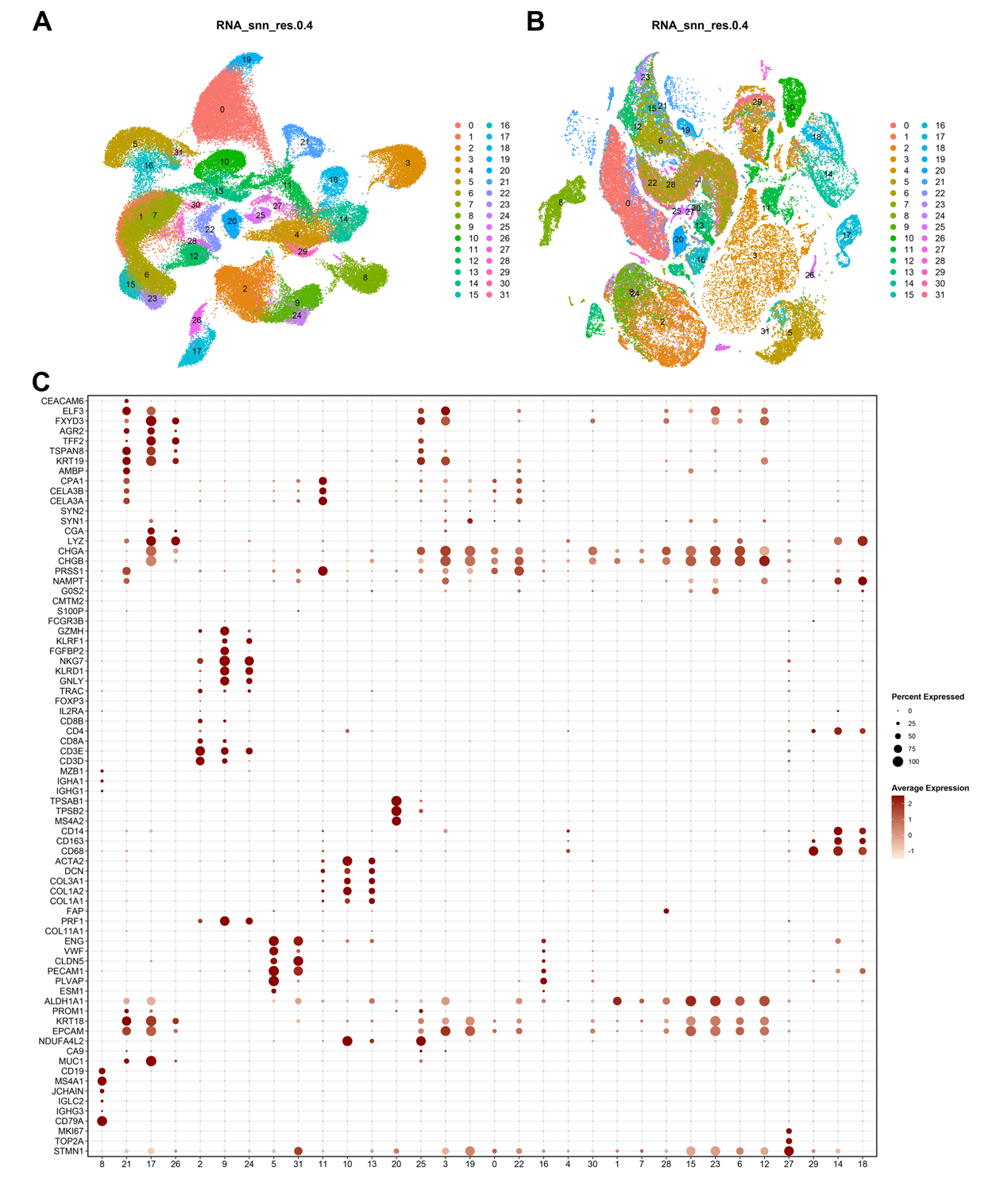


**Supplementary figure 2. Compartmentalization and subpopulation characterization information for single-cell sequencing data.**

1. The UMAP plot demonstrates each cell clustering in pancreatic neuroendocrine carcinoma.
2. The t-distributed stochastic neighbor embedding (t-SNE) plot demonstrates each cell clustering in pancreatic neuroendocrine carcinoma.
3. Dot plot exhibited all known markers collected from established literatures were used to annotate each subpopulation.

**Supplementary Figure 3**


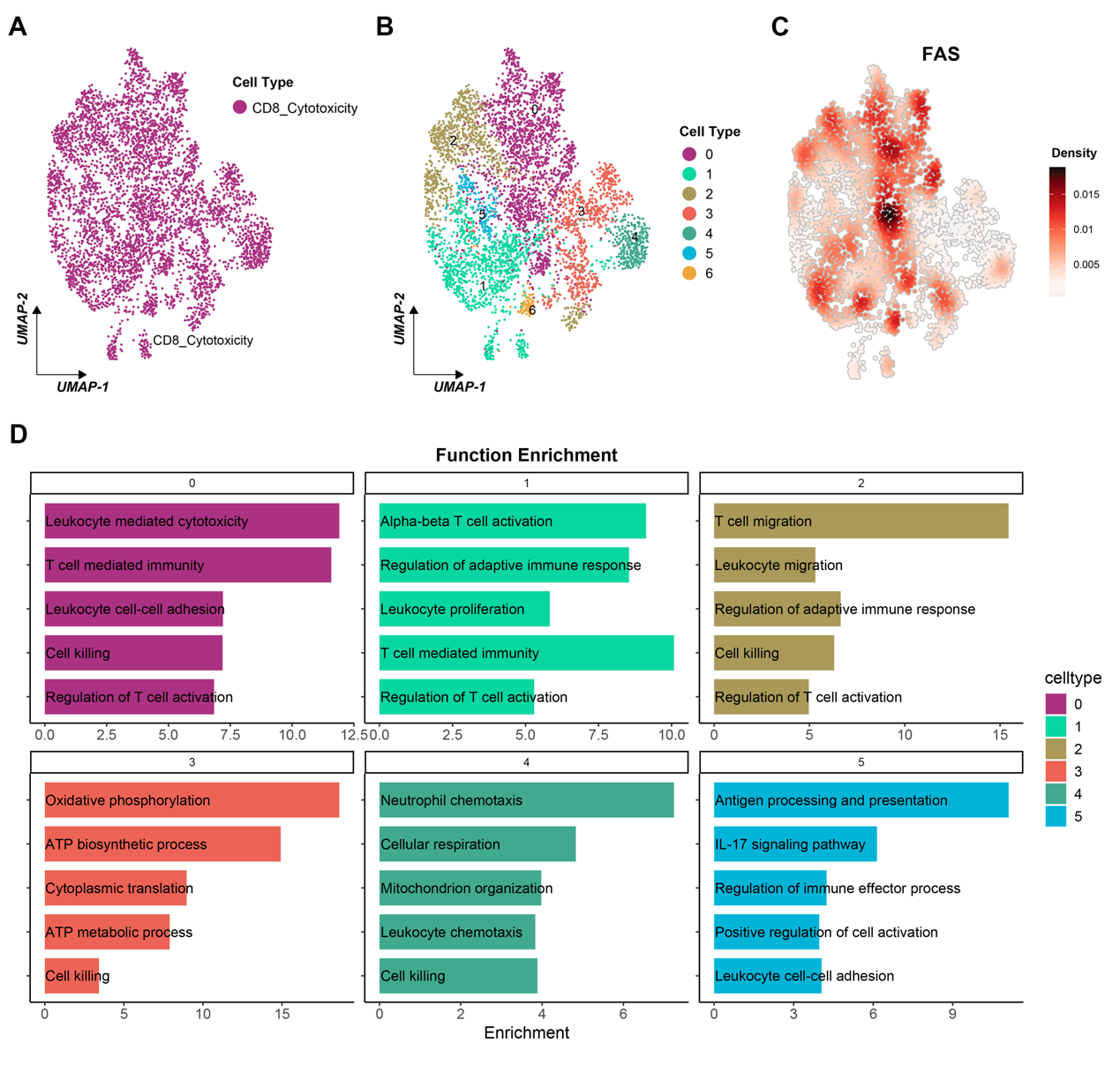


**Supplementary figure 3. Single-Cell transcriptomic profiling of CD8^+^ T cell subpopulations in PNETs.**

(A) and (B) UMAP visualization and proportional distribution of CD8^+^ T cell clusters. UMAP plot showing 7 distinct subpopulations (Clusters 0–6) identified by unsupervised clustering (Seurat v4.0, resolution=0.8) from CD8^+^ cells.

(C) Spatial mapping of Fas expression across CD8^+^ T cell clusters. Fas expression overlaid on the UMAP embedding (log-normalized counts).

(D) Functional enrichment of CD8^+^ T cell subpopulations. The bar chart quantifying the relative abundance of each cluster. Cluster proportions were calculated after excluding doublets and low-quality cells (mitochondrial genes < 20%).

**Supplementary Figure 4**


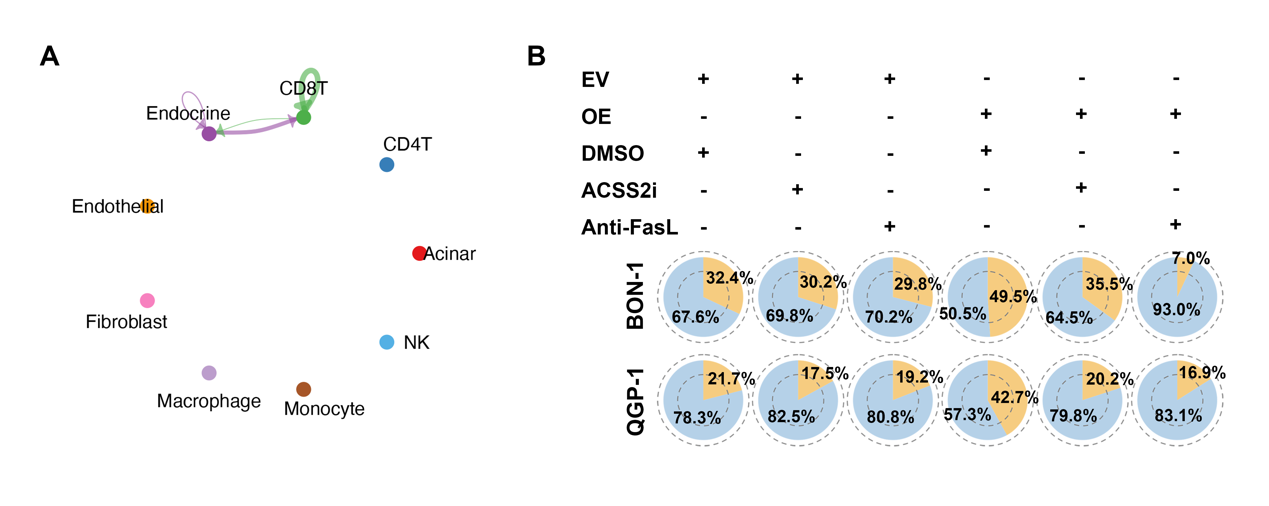


**Supplementary figure 4. Interaction of PNET cells with CD8^+^ T cell cells and pro-apoptotic effects on Jurkat cells.**

1. Intercellular communication network between CD8T cells and endocrine cells calculated from CellChat.
2. PNETs cells and Jurkat cells were co-cultured after treatment with different conditions and the percentage of Jurkat cells undergoing apoptosis was recorded. The addition of ACSS2i (0.5 μM) or FasL antibody (0.1 μg mL^-1^) to the ACSS2-EV group showed a tendency to reduce the proportion of apoptotic cells in Jurkat cells but did not induce significant changes. In the ACSS2-OE stable transfection group of BON-1 and QGP-1 cell lines, significant inhibition of apoptosis was observed in Jurkat cells after the addition of ACSS2i (0.5 μM) or FasL antibody (0.1 μg mL-1), especially in the group given FasL antibody (both *P* < 0.05).

**Supplementary Figure 5**


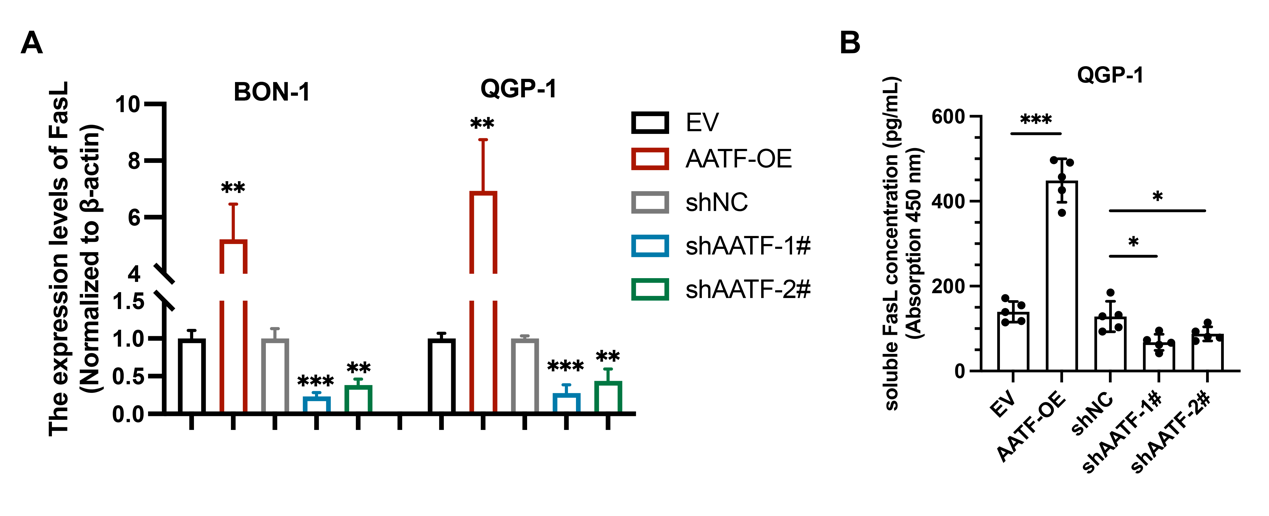


**Supplementary figure 5. Validation of expression levels of FasL modulated by AATF in PNET cells.**

1. Overexpression (or knockdown) of AATF in PNET cells could lead to increased (or decreased) mRNA levels of FasL. The *FasLG* gene expression levels were analyzed as verified by RT-qPCR assays in different groups of BON-1 and QGP-1 cell lines, respectively (** *P* < 0.01, *** *P* < 0.001).
2. Overexpression (or knockdown) of AATF in PNET cells could lead to increased (or decreased) sFasL levels. ELISA assay of sFasL concentration levels in QGP-1 cell culture supernatants from different treatment groups. Five independent repetitions for each group (**P* < 0.05, *** *P* < 0.001).

**Supplementary Figure 6**


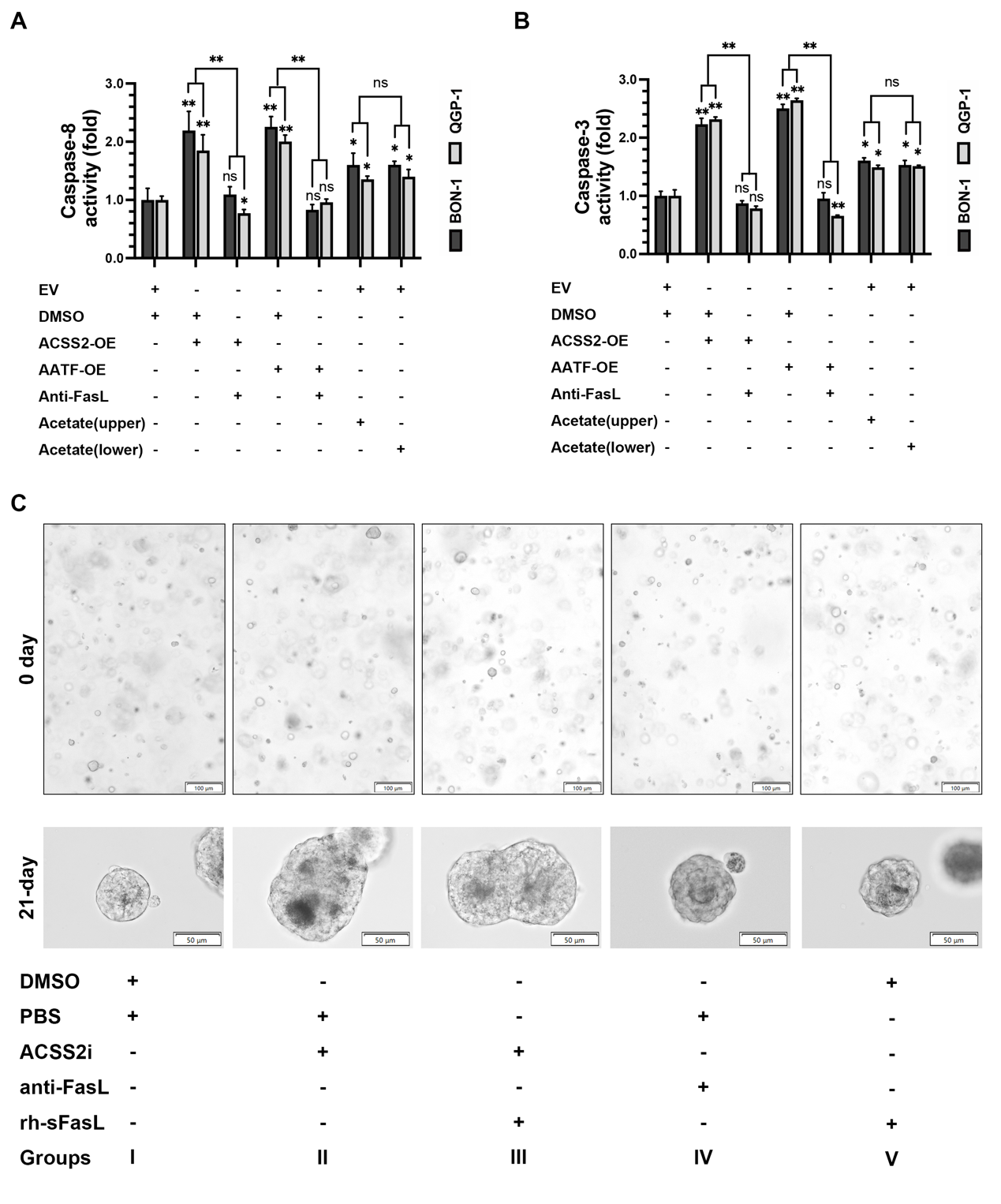


**Supplementary figure 6. ACSS2/AATF-driven sFasL activates caspase-8/3 in co-cultured Jurkat cells and modulates apoptosis in patient-derived organoids.**

**(A**, **B)** Changes in the activity of caspase-8 **(A)** and caspase-3 **(B)** in Jurkat cells after co-cultured with BON-1 and QGP-1 cells were determined by fluorescence assay after the corresponding treatments. Acetate (1 mM) was detected after 48 h of independent treatment in the upper and lower chambers of co-cultured six-well plates. FasL antibody (0.1 μg mL^-1^) was detected after 48 h of incubation in the lower chamber of co-cultured six-well plates (* *P* < 0.05, ** *P* < 0.01, ns indicates not significant).

**(C**) Representative brightfield images of patient-derived organoids (PDOs). The organoids were evenly inoculated in six-well plates and recorded for 0 days. Until the 21st day of normal culture, the PDOs were treated respectively and divided into Groups I ~ V. CD8^+^ T cells (1×10^5^/well) were inoculated in the transwell chamber and co-cultured indirectly with PDOs for 48 h. Scale bar: 100 μm (for 0 day) 50 μm (for 21-day).

**Supplementary Figure 7**


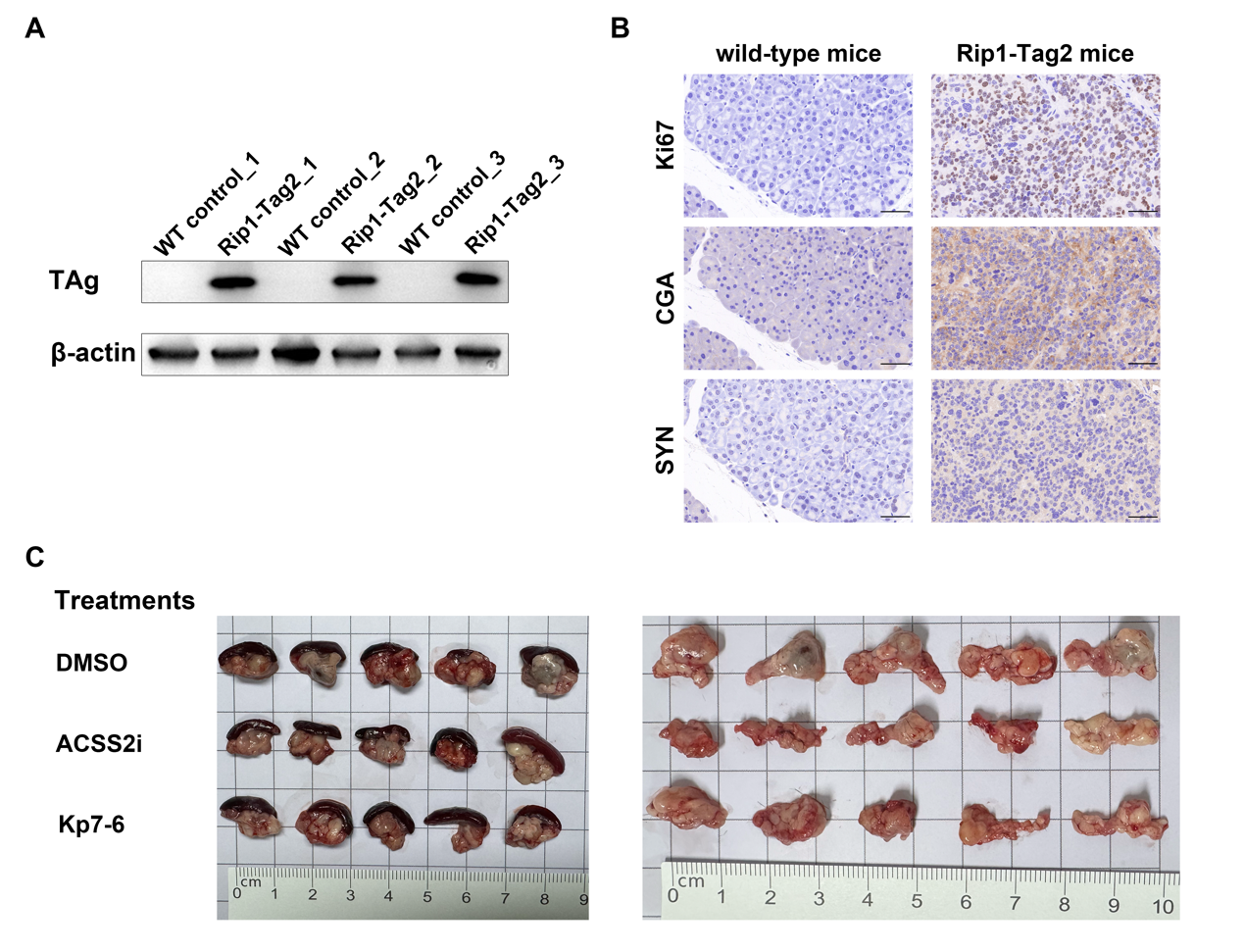


**Supplementary figure 7. The validation of Rip1-Tag2 transgenic mouse model and the photographed primary PNET and adjacent spleen tissues.**

**(A)** Western blotting demonstrating the expression level of the transgenic vector simian virus 40 large Tantigen gene (SV40 TAg) in the pancreatic islets of our developed Rip1-Tag2 mice.

**(B)** Representative images of IHC staining of ki67 and PNET pathological markers (CGA and SYN), for wild-type mice and Rip1-Tag2 mice in pancreas. Scale bars, 50 μm.

**(C)** The primary PNET and adjacent spleen tissues, as well as the primary PNET tissues of Rip1-Tag2 mice were collected and photographed (scale bar = 1cm, n = 5).
